# Hypochlorous Acid: From Innate Immune Factor and Environmental Toxicant to Chemopreventive Agent Targeting Solar UV-Induced Skin Cancer

**DOI:** 10.3389/fonc.2022.887220

**Published:** 2022-04-29

**Authors:** Jeremy A. Snell, Jana Jandova, Georg T. Wondrak

**Affiliations:** Department of Pharmacology and Toxicology, R.K. Coit College of Pharmacy & UA Cancer Center, University of Arizona, Tucson, AZ, United States

**Keywords:** hypochlorous acid, chlorination stress, environmental exposure, skin exposome, solar ultraviolet radiation, inflammation, skin cancer

## Abstract

A multitude of extrinsic environmental factors (referred to in their entirety as the ‘skin exposome’) impact structure and function of skin and its corresponding cellular components. The complex (i.e. additive, antagonistic, or synergistic) interactions between multiple extrinsic (exposome) and intrinsic (biological) factors are important determinants of skin health outcomes. Here, we review the role of hypochlorous acid (HOCl) as an emerging component of the skin exposome serving molecular functions as an innate immune factor, environmental toxicant, and topical chemopreventive agent targeting solar UV-induced skin cancer. HOCl [and its corresponding anion (OCl^-^; hypochlorite)], a weak halogen-based acid and powerful oxidant, serves two seemingly unrelated molecular roles: (*i)* as an innate immune factor [acting as a myeloperoxidase (MPO)-derived microbicidal factor] and (*ii*) as a chemical disinfectant used in freshwater processing on a global scale, both in the context of drinking water safety and recreational freshwater use. Physicochemical properties (including redox potential and photon absorptivity) determine chemical reactivity of HOCl towards select biochemical targets [i.e. proteins (e.g. IKK, GRP78, HSA, Keap1/NRF2), lipids, and nucleic acids], essential to its role in innate immunity, antimicrobial disinfection, and therapeutic anti-inflammatory use. Recent studies have explored the interaction between solar UV and HOCl-related environmental co-exposures identifying a heretofore unrecognized photo-chemopreventive activity of topical HOCl and chlorination stress that blocks tumorigenic inflammatory progression in UV-induced high-risk SKH-1 mouse skin, a finding with potential implications for the prevention of human nonmelanoma skin photocarcinogenesis.

## Introduction: Environmental Exposure and Skin Health: Focus on Solar Ultraviolet Radiation and Co-Exposure to Environmental Toxicants

Skin, the largest part of the human integumentary system constituting about 15% of the total adult body mass, is positioned at the interface between environment and the body’s internal organs (1). The skin is a crucial and dynamic barrier against the constantly changing environment, autonomously maintaining organ-level and systemic homeostasis. As one of the key barriers of defense against physical, chemical, and microbial stressors, the skin is a complex organ functioning in tissue regeneration and wound healing, hydro-, osmo-, and thermoregulation, endocrine and sensory functions, biosynthesis, metabolism, innate and adaptive immunity, circadian rhythmicity, and neuro-psychosocial communication (1–8). Among various environmental factors relevant to human health, solar exposure is known to impact tissue homeostasis modulating many of these cutaneous functions. Indeed, skin barrier dysfunction is a hallmark of numerous cutaneous pathologies including allergic reactions, microbial infection, photoaging, and photocarcinogenesis.

As an outer surface organ, human skin is ubiquitously exposed to solar ultraviolet (UV) radiation. UV exposure has both positive and negative effects on human health (9). It is responsible for the biosynthesis of vitamin D_3_, can stimulate the production of photoprotective melanin, and is used therapeutically to treat inflammatory skin diseases (such as psoriasis, vitiligo, localized scleroderma, and atopic dermatitis). At the same time, solar UV is a potent environmental human carcinogen (10–12). The mechanisms by which solar UV-radiation causes skin photodamage are wavelength-dependent (11). UVB (290-320 nm) is thought to cause direct structural damage to DNA in the form of epidermal cyclobutane pyrimidine dimers (CPDs) and other photoproducts. Most of the solar UV energy incident on human skin derives from the deeply penetrating UVA region (≥ 95%, 320-400 nm) not directly absorbed by DNA, and UVA-induced photodamage occurs by oxidative mechanisms mediated by reactive oxygen species (ROS). Contributing to the adverse effects of solar UV exposure is its known action as a systemic immunosuppressant, compromising an individual’s immune response with mechanistic implications for photocarcinogenesis (13).

**Figure 1:**
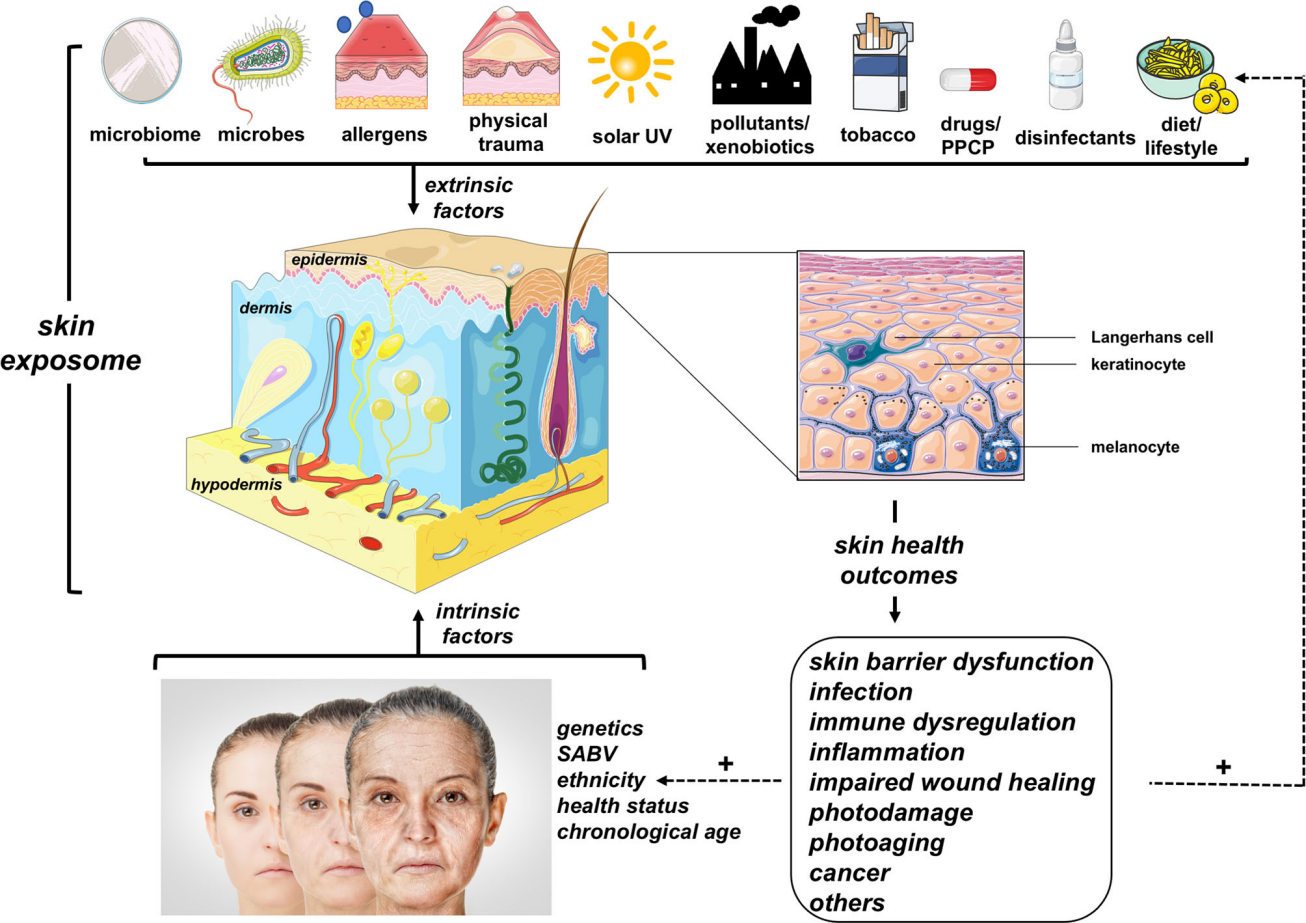
The Skin Exposome. A multitude of extrinsic environmental factors (referred to in their entirety as the ‘skin exposome’) impact structure and function of skin and its corresponding cellular components. The complex (i.e. additive, antagonistic, or synergistic) interactions between multiple extrinsic (exposome) and intrinsic (biological) factors are important determinants of skin health outcomes. Unfolding skin pathology can potentiate (+) the cutaneous vulnerability to further environmental exposures or intrinsic factors (fueling a positive feedback loop). (PPCP, pharmaceuticals and personal care products; SABV, sex as a biological variable). Image was created using free imaging software: smart.servier.com.

UV and other environmental toxicants can be conceptualized as components of the overall skin exposome (**Figure 1**), a term integrating all environmental cutaneous exposures and consequent biological effects including antagonism and potentiation that may result from co-exposures (14): (*i*) physical (such as thermal and mechanical trauma), (*ii*) chemical/xenobiotic [such as industrial pollutants, topical and systemic drugs, disinfectants, pharmaceuticals and personal care products (PPCPs)], (*iii*) microbiomic (originating from commensal and pathogenic microbes), (*iv*) allergenic (either of chemical or biological nature), and (*v*) life style-associated (such as tobacco product use, dietary choices, circadian rhythmicity, sleep pattern etc.) factors. Importantly, the complete skin exposome is subject to cross-talk with intrinsic factors (i.e. an individual’s primary biological determinants of skin structure and function) including: (*i*) genetics (as associated with ethnicity, sex as a biological variable (SABV), disease vulnerabilities etc.), (*ii*) pathobiological occurrences [such as infections, metabolic dysregulation (including diabetes), and autoimmune disturbances], and (*iii*) chronological aging (7, 15–20). Certain aspects and subcategories of the skin exposome have been expertly reviewed including the skin microbiome and the skin redoxome (7, 8).

Molecular crosstalk and mechanistic overlap between various components of the extrinsic skin exposome is well substantiated at the molecular level. For example, potentiation of solar UV-induced cutaneous and systemic injury by co-exposure to other environmental toxicants/pollutants has attracted much attention due to its negative impact on public health worldwide. Indeed, common environmental toxicants such as heavy metals (e.g. cadmium), metalloids (e.g. arsenic), and organic xenobiotics (e.g. benzo[a]pyrene, TCDD) are established potentiators of solar UV damage and skin carcinogenesis (21–23). Co-carcinogenicity of various exposome factors potentiating solar UV-induced skin photocarcinogenicity is firmly documented, as applicable to: (*i*) pollutants such as polyaromatic hydrocarbons including benz[a]pyrene (from cigarette smoke and combustion engines), (*ii*) arsenic (from drinking water), (*iii*) hypercaloric dietary intake/metabolic dysregulation, (*iv*) molecular therapeutics [acting as photosensitizers or immunosuppressants], (*v*) and microbial infection (HPV, Merkel cell polyoma virus, Malassezia spp.) (21–28). To the contrary, dietary intake of specific phytochemicals representing an extrinsic exposome-related factor might enhance skin barrier function and antagonize photo-carcinogenesis, acting through modulation of specific molecular pathways associated with enhancement of antioxidant stress response (with involvement of the Keap1/NRF2 pathway) and suppression of inflammatory signaling (NFκB and AP-1) (9, 29).

Likewise, impairment of skin barrier function and health can result from the overlap of extrinsic (exposome-related) and intrinsic factors that interact and potentially synergize in complex ways. For example, it is well documented that smoking (an external exposomal factor) accelerates skin aging (intrinsic factor) (30). Likewise, human skin photoaging represents the overlap of intrinsic factors (such as cellular senescence as a function of chronological age) and structural/functional alterations due to environmental solar exposure (31). In the context of co-carcinogenicity, it has long been known that intrinsic genetic alterations that impair DNA repair capacity are associated with an increased UV-induced skin cancer incidence as substantiated paradigmatically by xeroderma pigmentosum patients with excision repair deficiencies underlying a pronounced increase in skin cancer risk (32, 33).

Recently, hypochlorous acid (HOCl) has been identified as an environmental toxicant relevant to cutaneous exposures (34–36). Here, given the ubiquitous use of topical HOCl-based disinfection strategies combined with its established biological role as an essential determinant of neutrophil-related innate immunity, we review the role of this powerful electrophile as an understudied chemical component of the skin exposome with special emphasis on novel data that substantiate HOCl-dependent modulation of solar UV-induced skin carcinogenesis.

## Hypochlorous Acid and its Conjugated Anion: Innate and Environmental Mediators of Oxidant Chlorination Stress

### HOCl in Innate Immunity

**Figure 2:**
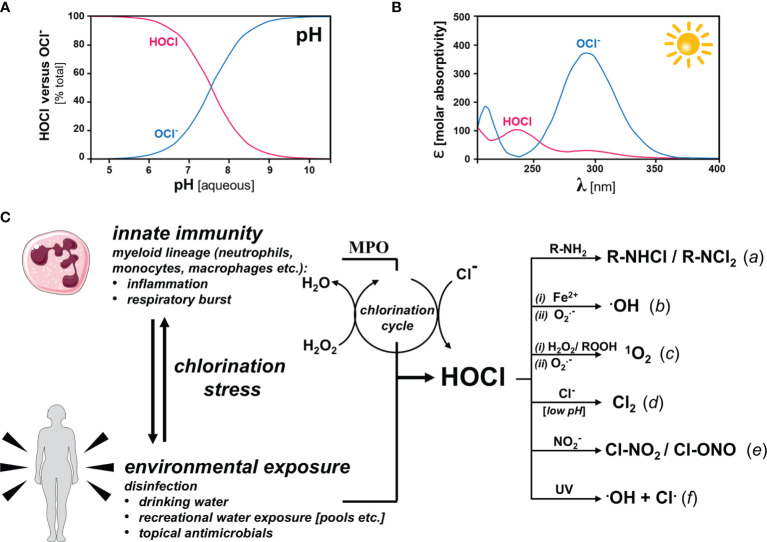
HOCl/OCl-: Physicochemical Properties, Innate and Environmental Origin, and Formation of HOCl-Derived Secondary Oxidants Under Physiological Conditions (**A**) pH-dependent speciation (HOCl versus OCl-). At physiological pH, HOCl and OCl- occur at near equimolar ratios **(B)** Photon Absorptivity. HOCl and its corresponding anion differ with regard to photo-absorptive properties: HOCl (λ_max_ = 235 nm; є = 101); OCl^-^ (λ_max_ = 292 nm; є = 365). OCl^-^ absorptivity covers the solar UVB (290-320 nm) and UVA-II (320-340 nm) regions. **(C)** Biological and environmental sources of HOCl formation and HOCl-derived secondary oxidants. Left panel: Innate immune activation causes HOCl production by specific myeloid cells under conditions of inflammation and respiratory burst *via* the myeloperoxidase (MPO)-catalyzed chlorination cycle that consumes H_2_O_2_ for Cl^-^ oxidation. Environmental exposure to HOCl occurs in the context of freshwater disinfection (e.g. drinking water, recreational use, etc.) and topical antimicrobial intervention. Right panel: HOCl-derived secondary oxidants. Apart from acting as potent oxidizing species, HOCl/OCl^-^ may be involved in a number of biochemically relevant reactions producing secondary oxidants including: (*a*) chloramine formation; (*b*) hydroxyl radical formation downstream of (*i*) Fe(II)-dependent Fenton or (*ii*) superoxide chemistry; (*c*) singlet oxygen formation downstream of (*i*) peroxide or (*ii*) superoxide chemistry; (*d*) molecular chlorine formation with involvement of Cl^-^ at low pH; (*e*) formation of nitryl chloride and chlorine nitrite upon reaction with nitrite; and (*f*) formation of hydroxyl and chlorine radicals as a result of UV-driven photolysis.

Basic physicochemical properties of HOCl are relevant to its endogenous physiological function including its role as an innate immune factor, topical antimicrobial, and environmental toxicant (**Figure 2**) (37, 38).

Importantly, multiple chemical parameters dictate the biological function of HOCl serving as an important component of the skin exposome. In this context it should also be mentioned that HOCl-dependent chlorination stress is dictated by both thermodynamic and kinetic parameters that ultimately determine susceptibility of various biochemical targets (39–43).

First, HOCl is (*i*) a weak acid, (*ii*) a powerful chlorination agent, and (*iii*) direct- or indirect-acting oxidant. HOCl contains one labile proton (pKa = 7.46) dictating the co-existence between acid and conjugated base under physiological conditions at near equimolar ratio (**Figure 2A**). Another important physico-chemical feature of HOCl and its corresponding anion [OCl^-^ (hypochlorite)], relevant to environmental co-exposure scenarios, is its ability to absorb solar UVB (290-320 nm) photons and, as a consequence, undergo photolysis (**Figure 2B**). HOCl maximally absorbs at 237 nm and 289 nm with molar extinction coefficients of 102 and 36.1, respectively; OCl^-^ maximally absorbs at 292 nm with a molar extinction coefficient of 378. Consequently, photolysis of HOCl by environmentally relevant UVB is a function of pH. Indeed, environmental UV exposure might cause photolysis reactions with formation of various reactive species including the hydroxyl and chlorine free radicals, among others. However, the specific role of photolysis in the mediation of biological HOCl-based chlorination stress remains to be explored, given the opposing effects of a short reactivity-limited lifetime and sustained HOCl-release from photostable organic precursors including chloramines (such as the swimming pool disinfectant trichloroisocyanuric acid; see **Figure 5A**, structure 5) (44, 45).

Remarkably, HOCl, a weak halogen-based acid and powerful oxidant, serves two seemingly unrelated molecular roles: (*i)* as an innate immune factor [acting as a myeloperoxidase (MPO)-derived microbicidal factor] and (*ii*) as a chemical disinfectant used in freshwater processing, both in the context of drinking water safety and recreational use (e.g. swimming pool/hot tub disinfection) (37, 38). Importantly, HOCl and its conjugated base represent a potent oxidizing redox system [E^0^’ = +0.9 (OCl^-^); E^0^’ = +1.48 V (HOCl)] under physiological conditions. In this context, it is important to notice that the major anti-microbially active species is thought to be HOCl (compared to the hypochlorite anion), consistent with the half-cell oxidation-reduction potentials and an increased ability of the uncharged HOCl species to penetrate cell walls and membranes of pathogens. Involvement of MPO in antimicrobial response and host pathogen interaction have been covered elsewhere and will not be the topic of this review (46). The potent oxidant HOCl/OCl^−^ serves as an endogenous microbicidal agent, generated by myeloid lineage-derived effector cells (including neutrophils). Indeed, during the respiratory burst, MPO-dependent oxidation of chloride anions (using NADPH oxidase-derived superoxide/hydrogen peroxide) produces HOCl and other hypohalous acids such as HOBr (hypobromous acid), HOI (hypoiodous acid), and HOSCN (hypothiocyanous acid)] as an essential component of antimicrobial innate immunity (**Figure 2C**) **(** 47, 48). The ‘chlorination cycle’ catalyzed by MPO involves the hydrogen peroxide-dependent oxidation of reactive site ferric iron [Fe (III)] forming a highly reactive oxy-ferryl [Fe(IV)=O] radical cation capable of oxidizing chloride anions leading to the formation of HOCl and regeneration of the ferric iron MPO. Importantly, endogenous hypohalous acids, even though serving innate host defense functions, may also induce tissue damage at sites of inflammation, an area of active research in the context of neurodegenerative disease (M. Alzheimer; M. Parkinson), metabolic and cardiovascular dysfunction (atherosclerosis; diabetes), autoimmune dysregulation, cancer, and chronological aging, among others (47, 49, 50). Importantly, beyond a role in cutaneous innate immunity, the MPO system has also been involved in various skin pathologies, either serving as a causative factor or biomarker in inflammation, contact hypersensitivity and irritation, psoriasis, UV-damage, photoaging, and carcinogenesis (51–59).

### HOCl in Freshwater Disinfection: From Human Consumption to Recreational Use

The disinfection of drinking water supply by HOCl-dependent chlorination may well be regarded as the most important public health milestone in human history. Among the sustainable development goals adopted by members of the United Nations in 2015 is goal 6, which aims to provide all people with equal access to safe and affordable drinking water, sanitation and hygiene as consistent with the 2010 proclamation of the general assembly that such encompasses a human right. Despite substantial progress, it is currently estimated that more than 2 billion people lack access to safely managed drinking water and basic hygiene, while nearly half of the human population lacks sanitation. Indeed, according to global population projections and climate change models, supply problems surrounding safe water will be of utmost importance for this century. Considering these trends, continual optimization of the methods for drinking water sanitation, distribution, safe storage and wastewater treatment will be necessary to reduce water related health disparities on a global scale (60).

### HOCl-Based Swimming Pool Disinfectants: Oxidative Potentiators of Cutaneous Solar UV Damage as an Unexplored Environmental Exposure of Global Importance

HOCl is the active microbicidal principle released by standard swimming pool disinfectants employed abundantly worldwide. According to CDC, there are 10.4 million residential and 309,000 public swimming pools and over 7.3 million hot tubs in the United States alone (https://www.cdc.gov/healthywater/swimming/fast-facts.html). Even though HOCl, commonly referred to as ‘swimming pool chlorine’, is the most frequently used halogen-based oxidizing pool disinfectant, little research has addressed toxicological implications and damage potentiation resulting from combined exposure to HOCl-based swimming pool disinfectants and solar UV as it occurs on a global scale in the context of recreational swimming pool use (34). Pool disinfection is an essential barrier to the spread of germs. To ensure a non-infectious healthy pool environment, operators try to maintain a desired range (1.0-1.5 ppm free HOCl; for outdoor swimming pools and indoor pools smaller than 20 m^2^, the recommended maximum level is 5 ppm). In recent years, use of sodium dichloroisocyanurate, an organic HOCl-precursor, has gained frequent use, but HOCl/OCl^-^ is the predominantly active microbicidal agent (34, 61).

Human skin is extensively exposed to HOCl-based pool disinfectants causing oxidation and chlorination of specific molecular targets; however, little molecular research exploring the potentially adverse cutaneous and systemic effects resulting from exposure to HOCl-disinfectants during recreational swimming pool use has been conducted. Given the important role of photo-oxidative mechanisms underlying adverse cutaneous effects of solar UV exposure and the largely oxidative nature of chlorination-induced damage, it seems reasonable to expect synergistic molecular interactions that drive HOCl-potentiation of sun damage in exposed individuals. Indeed, according to the recent *WHO Guidelines for Safe Recreational Water Environments*, epidemiological evidence indicates that risk of sunburn and cutaneous photodamage is increased in swimming pool environments.

In addition to direct target chlorination and oxidation, HOCl-dependent reactions of biological relevance in inflammation and antimicrobial defense (-also observed in the context of topical disinfectant use-), might be mediated through the formation of numerous HOCl-derived electrophilic species (**Figure 2C**; right portion). Chloramine formation involves the HOCl-dependent derivatization of primary and secondary biological amines as contained in small biochemicals (such as histamine and taurine) and macromolecules (proteins etc.) (62–64). Moreover, hydroxyl radical formation may occur downstream of either Fe(II)-dependent Fenton chemistry, scenarios observable under conditions of MPO-facilitated heme degradation as a consequence of excess HOCl formation or pathological elevation of labile iron (65–67). Likewise, hydroxyl radicals can form upon reaction of HOCl with superoxide free radicals (68). Interestingly, HOCl-dependent formation of highly reactive photoexcited molecular oxygen [^1^O_2_ (singlet oxygen)] has been documented without mechanistic involvement of photons downstream of peroxide (including linoleic acid hydroperoxide), superoxide, or chloramine chemistry involving the chemical formation of photo-excited states (commonly referred to as ‘chemiexcitation’) (69–71). Molecular chlorine (Cl_2_) is another species formed downstream of MPO-dependent transformation of Cl^-^ anions and hydrogen peroxide at low pH, relevant to cholesterol chlorination in atherosclerotic pathology (72–74). Furthermore, upon reaction with nitrite, formation of nitryl chloride and chlorine nitrite might occur, reactions relevant to inflammatory protein nitration (75). Lastly, as a result of UV-driven photolysis generation of hydroxyl and chlorine radicals has been documented, a reaction of potential relevance to environmental co-exposure scenarios where solar photons in the UVB range might cause HOCl/OCl^-^ degradation with formation of reactive free radical species consistent with the extensive UVB absorptivity of OCl^-^ (38).

## Biomolecular Targets of Chlorination Stress: From Chemical Modification to Pathophysiological Consequences

Chlorination stress that occurs under physiological or environmental exposure-relevant conditions impacts structure and function of numerous classes of biomolecules, either through covalent introduction of chlorine (and chlorine-derived substituents) or through indirect oxidative insult. HOCl, in equilibrium at physiological pH with its anionic form [hypochlorite (OCl^−^)], may also induce tissue damage at sites of inflammation involving the oxidation and chlorination of biomolecules targeting peptides (e.g. glutathione), proteins, lipids, and nucleic acids (39, 42, 43, 47, 76, 77).

**Figure 3:**
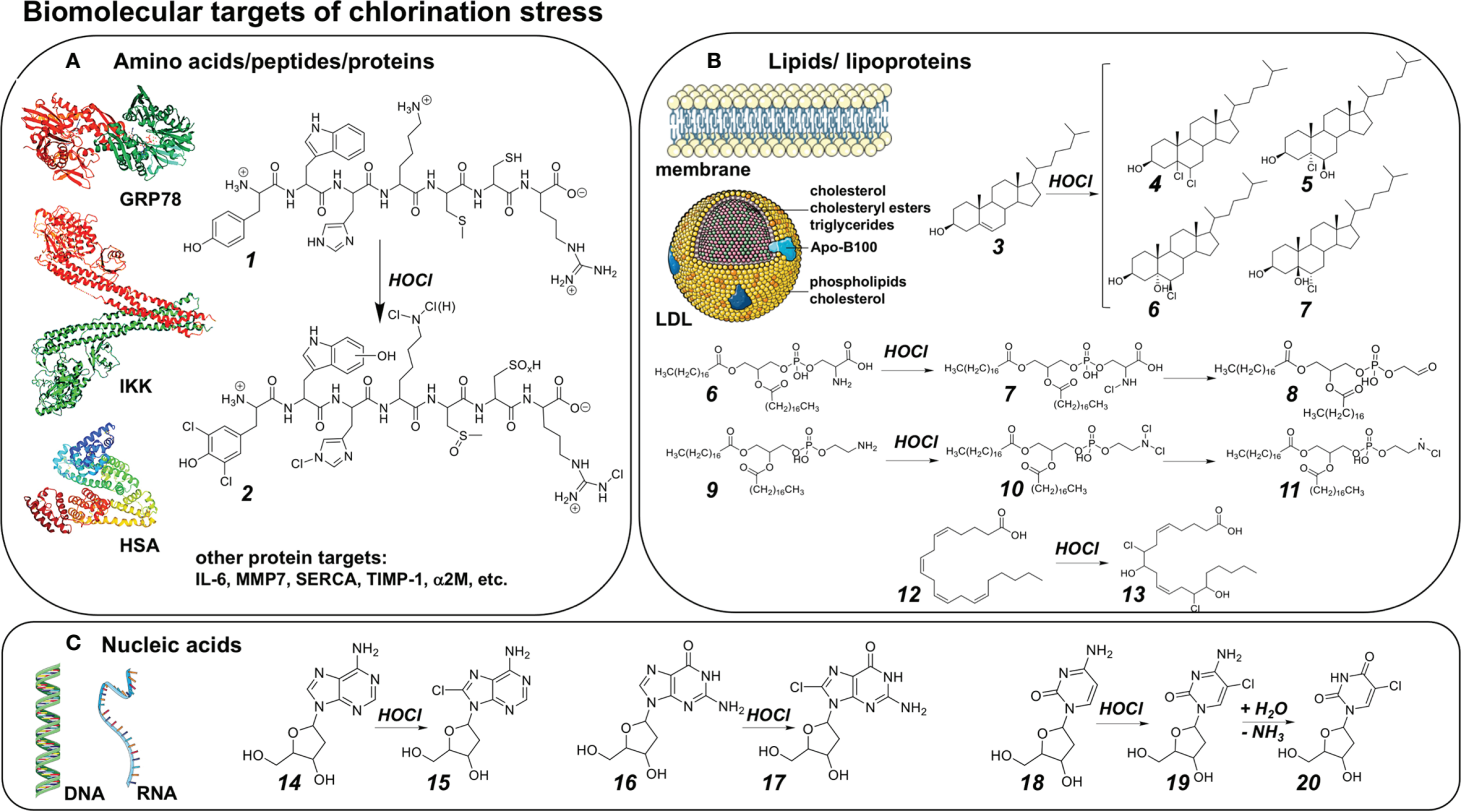
Biomolecular Targets of Chlorination Stress **(A)** Amino acids, peptides, and protein targets of chlorination stress. Theoretical heptapeptide [H_2_N-Tyr-Trp-His-Lys-Met-Cys-Arg-COOH (1)] illustrating the range of possible amino acid modifications (2) induced by HOCl (from amino- to carboxyterminus): Dichloro-tyrosine, hydroxy-tryptophan, histidine chloramine, lysine mono- or dichloramine, methionine sulfoxide, cysteine sulfenic/sulfinic/sulfonic acid, arginine chloramine. **(B)** Fatty acids, lipids, and lipoproteins as targets of chlorination stress. HOCl-mediated modification of cholesterol (3) forms a number of cholesterol chlorohydrin stereoisomers: 5,6-dichloro cholesterol (4); (5R,6R)-5-chloro-6-hydroxy cholesterol (5); (5R,6R)-6-chloro-5-hydroxy cholesterol (6); (5S,6S)-6-chloro-5-hydroxy cholesterol (7), among others. For phospholipids, HOCl may either exert its effects near the head group, or at sites of unsaturation: HOCl-mediated modification of phosphatidylserine (6) results in phosphatidylserine chloramine (7), with further oxidation/decarboxylation to phosphatidyl glycoaldehyde (8). HOCl-mediated modification of phosphatidylethanolamine (9) results in the respective dichloramine (10) subsequently forming N-centered radicals acting as long-lived mediators. For simplicity, for both phospholipids each lipid moiety is stearate. Fatty acids possessing greater degrees of unsaturation are more prone to modification by HOCl: HOCl-mediated modification of arachidonic acid (12) results in the formation of arachidonic acid chlorhydrins such as 8,14-dichloro-9,15 dihydroxy arachidonic acid bis-chlorohydrin (13). **(C)** Nucleic acids as targets of chlorination stress. HOCl may modify DNA, RNA, and free nucleobases. (For simplicity, only HOCl-mediated modification of deoxyribosides is shown.) HOCl-modification of deoxyadenosine (14) forms 8-chlorodeoxyadenosine (15). HOCl-modification of deoxyguanosine (16) forms 8-chlorodeoxyguanosine (17). HOCl-modification of deoxycytidine (18) forms 5-chlorodeoxycytidine (19), followed by spontaneous deamination forming 5-chlorodeoxyuridine (20).

Previous research has identified key molecular modifications downstream of chlorination stress targeting amino acids, peptides, and proteins as dominant targets of biologically-relevant chlorination stress (**Figure 3A**). For illustration, a hypothetical heptapeptide [H_2_N-Tyr-Trp-His-Lys-Met-Cys-Arg-COOH] has been envisioned that exemplifies the range of possible amino acid modifications induced by HOCl exposure including dichloro-tyrosine, hydroxy-tryptophan, histidine chloramine, lysine mono- or dichloramine, methionine sulfoxide, cysteine sulfenic/sulfinic/sulfonic acid, and arginine chloramine (78). Protein chlorination has been associated with structural changes of target proteins including fragmentation, crosslinking, aggregation, unfolding, and modulation of specific functions such as immunogenicity, enzymatic activity and ligand-receptor interaction (48, 79). Numerous proteins are subject to chlorination stress-induced modulation through chemical changes under physiological conditions, including plasma proteins [e.g. HSA, alpha2M], histones, heat shock/ER stress response mediators and calcium signaling components (e.g. GRP78, SERCA), inflammatory signaling molecules (e.g. IL-6, IKK) and mediators of tissue remodeling (e.g. MMP7, TIMP-1), causing effects that are mostly consistent with modulation, attenuation, and resolution of inflammatory tissue responses (35, 80–89). Specifically, inactivation of IKK (inhibitor of IκB kinase) through oxidation (Cys114/115) is thought to cause the hypochlorite-dependent attenuation of psoriasis observable upon topical application (35). Similarly, GRP78 (glucose-regulated protein 78, HSPA5) modification through chloramine adduction (Lys 353) has been suggested to modulate autophagy and apoptosis in A549 lung cancer cells, and N-chlorination of HSA (human serum albumin) converts plasma proteins into efficient activators of the phagocytic respiratory burst (46, 86). In addition, biogenic amines, mostly through chloramine formation, have been demonstrated to serve as biomolecular targets of chlorination stress including histamine, serotonin, melatonin, and taurine among others (90, 91).

Consistent with chlorination-associated electrophilic stress, unsaturated lipids serve as major HOCl-targets under physiological conditions (**Figure 3B**) (92–99). Indeed, free fatty acids, triglycerides, phospholipids, as well as cholesterol and its derivatives, have all been validated as being susceptible to chemical modification under conditions of physiological or environmental chlorination stress conditions (**Figure 3B**). For example, HOCl-mediated modification of cholesterol forms a number of cholesterol-chlorohydrin stereoisomers as depicted; in addition, phospholipids may undergo derivatization at nitrogen-containing head groups (forming the respective chloramine) or at sites of unsaturation, followed by further oxidation/decarboxylation and N-centered free radical formation. In addition, other biochemical lipid mediators including plasmalogens, prostaglandins, and leucotrienes, involved in tissue remodeling and inflammatory signaling, have been shown to be subject to HOCl-dependent adduction with consequent alteration of signaling properties (98).

Nucleic acids are important targets of chlorination stress with possible mutagenic, genotoxic, and cytotoxic outcomes downstream of chemical modification (**Figure 3C**) (39, 100–102). Specifically, it is well documented that HOCl exposure causes chemical modification of DNA and RNA (and their respective nucleotides, nucleoside, and free nucleobases, irrespective of ribose- or deoxyribose- substitution). For example, HOCl-modification of deoxyadenosine forms 8-chlorodeoxyadenosine, and HOCl-modification of deoxyguanosine forms 8-chlorodeoxyguanosine. Interestingly, HOCl-modification of deoxycytidine forms a 5-chlorodeoxycytidine-intermediate, followed by spontaneous deamination forming stable 5-chlorodeoxyuridine causing miscoding damage downstream of chlorination stress. Indeed, chloro-derivatives of nucleic acids and their constitutive bases, apart from their functional involvement in mutagenic events, may also play an important yet underappreciated role as biomarkers of chlorination stress characteristic of specific pathological conditions.

## Endogenous, Phytochemical, and Synthetic HOCl-Antagonists: Antioxidants and Quenchers

**Figure 4:**
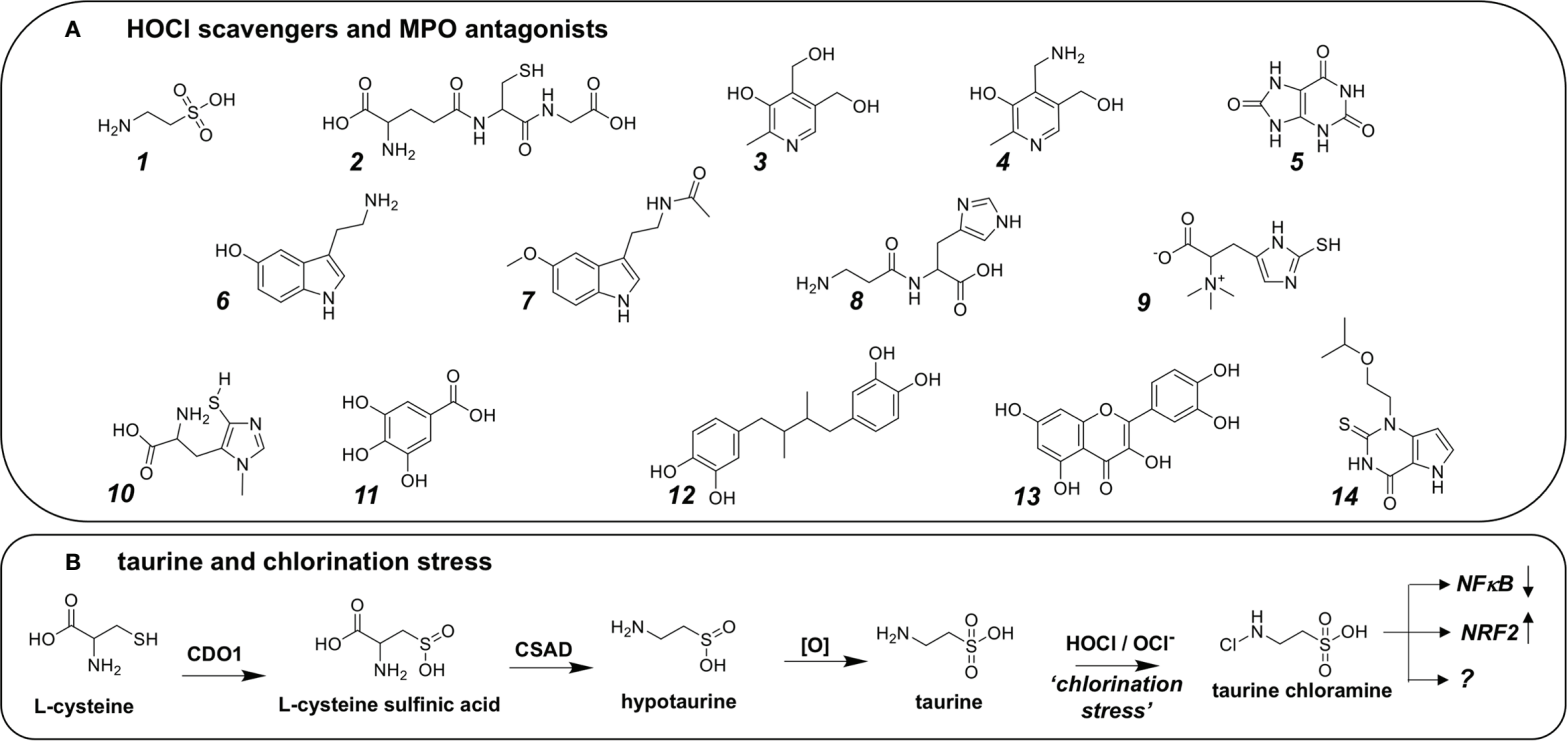
Biochemical, Natural Product, and Synthetic Hocl Scavengers and MPO Antagonists. **(A)** Endogenous chemical entities: 1. Taurine, 2. Glutathione, vitamins: 3. Vitamin B_6_, 4. Pyridoxamine; neurotransmitters: 6. Serotonin, 7. Melatonin 8. Carnosine; 9. Ergothioneine. Natural products: 10. Ovothiol, 11. Gallic acid, 12. Nordihydroguaiaretic acid, 13. Quercetin. Endogenous and synthetic MPO antagonists: 5. Uric acid, 7. Melatonin, 14. AZD3241 (Verdiperstat). **(B)** Endogenous production of taurine as a possible sink for HOCl. First, L-cysteine is oxidized by cysteine dioxygenase (CDO1) to form L-cysteine sulfinic acid, which in turn is decarboxylated by the enzyme cysteine sulfinic acid decarboxylase (CSAD) forming hypotaurine. Hypotaurine may undergo spontaneous oxidation to form taurine, acting as a sacrificial quencher of HOCl/OCl^-^ forming taurine chloramine as a moderately active chloramine with attenuated chlorination reactivity. Interestingly, taurine chloramine has been demonstrated to exert control over downstream signaling pathways including downregulation of NFκB, and upregulation of KEAP1-NRF2. As such, it has been hypothesized that taurine chloramine acts through posttranslational modification of distinct amino acid residues on transcription factors, among other effects.

Numerous molecular entities of endogenous or phytochemical origin have been shown to antagonize chlorination stress that occurs as a consequence of exposure to HOCl including amino acid derivatives (taurine, glutathione, serotonin, serotonin, carnosine, ovothiol, ergothioneine), phenolics (gallic acid, nordihydroguaiaretic acid, quercetin), and B_6_ vitamers (pyridoxal, pyridoxine, and pridoxamine), attributed mostly to chemical reactivity (i.e. sacrificial quenching) (**Figure 4A**). In addition, antagonists of MPO enzymatic activity (such as the synthetic MPO inhibitor verdiperstat or the endogenous metabolite uric acid) blocking HOCl formation have been explored for pharmacological control of pathophysiological chlorination stress (47, 62, 91, 103–109).

Among these biomolecules, B_6_-vitamers deserve special recognition since they have been shown to exert protection against chlorination stress as assessed using *in vivo* disease models, an effect attributed to formation of stabilized chloramine derivatives (110). Likewise, imidazole-derivatives (e.g. L-histidine, carnosine, carcinine) and thio-imidazole-derivatives (ergothioneine and sea urchin-derived ovothiol) have been identified as potent chlorination stress inhibitors (111–113).

Moreover, the cysteine-derived metabolite taurine (2-amino-ethane-sulfonic acid) has now been identified as a major endogenous HOCl-directed scavenger and antioxidant, attenuating physiologically relevant chlorination stress (**Figure 4B**). Strikingly, neutrophils represent a large reservoir of free taurine compromising approximately 50% of the cellular amino acid/amino acid-derivative pool thought to be involved in direct chemical protection against cytotoxic consequences of the respiratory burst associated with microbicidal HOCl formation (114). Taurine formation occurs as the result of enzyme-catalyzed cysteine transformation through intermediate generation of L-cysteine sulfinic acid and hypotaurine (**Figure 4B**). The consequent formation of N-chlorotaurine, representing a chlorinated adduct with attenuated reactivity, has also been interpreted as an intermediate step facilitating the extension of the phagocytic activity range, enabling enhanced stability and diffusion, spatially amplifying the range of oxidative antimicrobial effects. Indeed, attenuated chlorination reactivity of N-Chlorotaurine has been attributed to sulfonic acid-dependent electrostatic anionic shielding of the adjacent chloramine function that is amenable to chloro-transfer if attacked by biomolecular nucleophiles (115).

Importantly, N-chlorotaurine formation may cause the negative regulation of inflammatory processes by multiple distinct molecular mechanisms attenuating NF-kB and related cytokine signaling (88, 116). Interestingly, taurine might not only attenuate direct chemical reactivity of HOCl through sacrificial quenching, but chloro-taurine may then act as a redox-directed signaling modulator of major inflammatory targets and pathways. Indeed, it has been shown that N-chlorotaurine modulates inflammatory pathologies attributed to chemical modification of inflammatory factors, such as IL-6 and NFkB. Indeed, N-chlorotaurine exposure of IL-6 causes oxidation of residues relevant to IL6R receptor-binding (Met161 and Trp157) (88). Negative modulation of NF-κB by N-chlorotaurine (and other chloramines such as glycine chloramine) is thought to originate from oxidation of Met45 in IκB (preventing its ubiquitination and proteasomal degradation) (116, 117). Importantly, NRF2, the master transcriptional regulator of cellular antioxidant responses, has also been shown to be responsive to N-chlorotaurine-mediated chlorination stress, an effect attributed to electrophilic adduction and inactivation of Keap-1, the redox-sensitive negative regulator of this transcription factor (118).

## Molecular Mediators, Signaling Pathways, and Human Target Organs of Chlorination Stress

**Figure 5:**
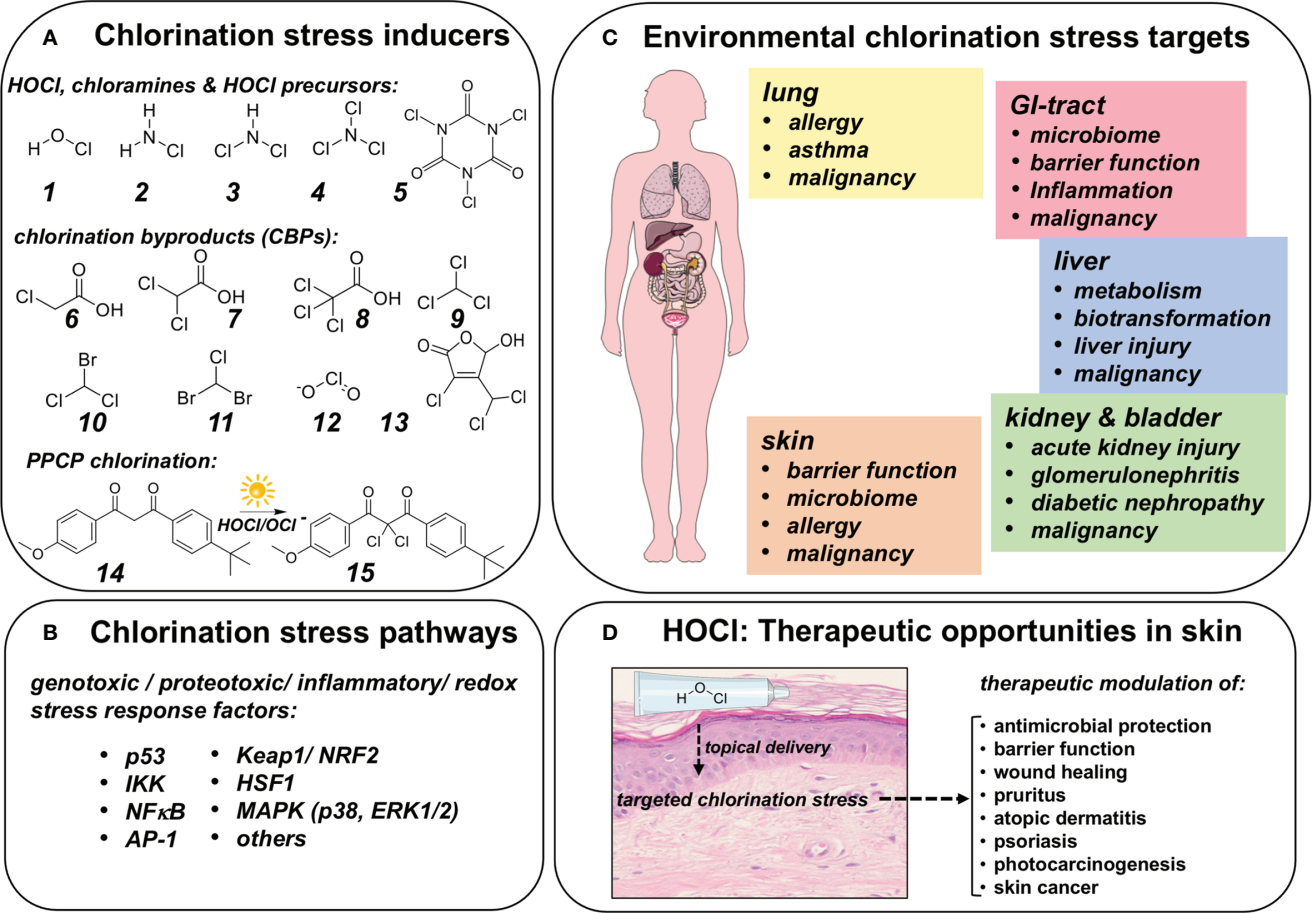
Chlorination Stress: Molecular Inducers, Signaling Pathways, Human Target Organs and Therapeutic Opportunities In Skin. **(A)** Direct and indirect chlorination stress inducers. 1. Hypochlorous acid, 2. Monochloramine, 3. Dichloramine, 4. Nitrogen Trichloride, 5. Trichloroisocyanuric acid. Upon chlorination of fresh water, chlorination byproducts (CBPs) are formed due to the presence of dissolved organic matter: Haloacetic acids: 6. Chloroacetic acid, 7. Dichloroacetic acid, 8. Trichloroacetic acid; Trihalomethanes: 9. Trichloromethane, 10. Bromodichloromethane, 11. Chlorodibromomethane, 12. Chlorite, all of which are subject to governmental regulation. Remarkably, numerous major chlorinated byproducts remain largely unexplored (and unregulated) such 3-chloro-4-(dichloromethyl)-5-hydroxy-5*H*-furan-2-one (13, commonly referred to as ‘mutagen X’). Additionally, pharmaceuticals and personal care products (PPCPs) introduced into the water supply are subject to HOCl mediated chlorination. As shown, the common UVA-sunscreen avobenzone (14) is chlorinated to produce a dichloro-species (15). **(B)** Chlorination stress signaling pathways. It has been demonstrated that chlorination stress may impact genotoxic, proteotoxic, inflammation and redox responses including p53, Keap1/NRF2, IKK/NFκB, and AP-1. **(C)** Human target organs of chlorination stress. Chlorination stress impacts multiple organ systems causing specific functional outcomes as discussed. **(D)** HOCl: Therapeutic opportunities in skin. HOCl may be used as a topical agent for therapeutic induction of chlorination stress in the context of antimicrobial intervention, impaired barrier function, wound healing, pruritus, atopic dermatitis, psoriasis, skin cancer, and prevention of photocarcinogenesis.

Molecular chlorination stress relevant to human health originates from HOCl (among other endogenous hypohalous acids including HOI and HOBr, formed mostly in the context of innate immunity) and is complemented by exposure to HOCl (and related derivatives) originating from exogenous sources. Specifically, environmental exposure-relevant chlorination agents include hypochlorous acid (and its corresponding anion) as well as diverse chloramines (e.g. monochloramine, dichloramine, nitrogen trichloride, chlorinated isocyanuric acid-derivatives) formed as a result of freshwater chlorination (**Figure 5A**) (119, 120). Interestingly, trichloroisocyanuric acid as well as its di-chloro-analogue are EPA-approved under FIFRA (Federal Insecticide, Fungicide, and Rodenticide Act) regulations, used globally for drinking water and freshwater disinfection (such as in swimming pools), offering increased photostability and sustained HOCl release (44, 121). Importantly, chlorination byproducts (CBPs) including organohaloacetic acids and trihalomethanes (formed due to the presence of dissolved organic matter) and chlorite are subject to strict EPA regulation due to potential adverse health effects (122, 123). Strikingly, out of more than six hundred halogenation byproducts identified as of to date, only eleven are currently subject to strict EPA regulation (124). For example, mutagen X (3-chloro-4-(dichloromethyl)-5-hydroxy-5*H*-furan-2-one) is a disinfection byproduct derived from humic acids, not regulated by EPA, with suspected involvement in cancer risk elevation associated with consumption of chlorinated drinking water, an effect attributed to genotoxicity surpassing that of currently regulated CBPs (including chloroform and bromodichloromethane) (125). Additionally, PPCPs introduced into the water supply are subject to HOCl-mediated chlorination and subsequent formation of CBPs. For example, common drugs including metformin, diclofenac, and tamoxifen entering freshwater sources are subject to direct chlorination causing drinking water contamination associated with largely unexplored implications for human health (126–129). Likewise, chlorination of PPCPs including sunscreen ingredients such as the common UVA-sunscreen avobenzone are associated with formation of a dichloro-species, and cosmetics are equally subject to chlorination with unexplored effects on human health (16, 130–135).

### Human Target Organs of Environmental Chlorination Stress

Importantly, human organ dysfunction may occur as a result of chlorination stress originating from exogenous (environmental) and endogenous (innate) sources (47, 49). Indeed, these pathophysiological outcomes have been attributed to the molecular consequences of chlorination stress (mediated through HOCl/OCl^-^ and HOCl-derived organic chloramines) impacting genotoxic, proteotoxic, inflammatory, and redox stress responses involving modulation of crucial transcription factor systems including p53, Keap1/NRF2, HSF1, IKK/NFκB, and AP-1 (**Figure 5B**) (35, 36, 118, 136–138). Likewise, signaling cascades including MAPKs (p38, ERK1/2) are sensitive to HOCl exposure attributed in part to tyrosine phosphatase modulation through cysteine-oxidation (139, 140). Also, in the context of balancing HOCl-related organ toxicity and therapeutic effects, it should be mentioned that the indiscriminate HOCl-dependent induction of chlorination stress might be associated with adverse irritant effects (51, 141–144).

Here, we will briefly focus on organ-specific toxicity of environmental exposure-induced chlorination stress (**Figure 5C**). In the lung, exposure to chlorination stressors has long been associated with a role in chronic inflammatory diseases of the respiratory system (137, 144–148). For example, competitive swimmers have been shown to suffer from high rates of asthma and airway hyperresponsiveness attributed to HOCl and volative DBP exposure (149, 150). In the context of pulmonary exposure, it is noteworthy that inhalational HOCl formulations are now undergoing clinical trials for prophylaxis and treatment of COVID-related respiratory infectious illness (*ClinicalTrials.gov Identifier: NCT04684550*). Moreover, there are concerns that innate or environmental chlorination stress might be related to the occurrence of lung malignancy related to genotoxic effects (86, 151, 152). Likewise, in the gastrointestinal tract, chlorination-associated changes have been substantiated, potentially impacting microbiome and barrier function, occurrence and severity of inflammatory pathology, and malignant progression (153–157). Hepatic toxicity related to chlorination stress, particularly in the context of environmental exposure to chlorination byproducts, has been documented extensively. Hepatic metabolism, biotransformation of drugs and xenobiotics have been investigated, and liver injury as well as malignancy have been substantiated as pathological outcomes resulting from chronic and dysregulated chlorination stress that might be potentiated by synergistic co-exposure involving multiple chlorinated chemical entities (158–161). Nephrotoxity and urogenital tract dysfunction are established pathological outcomes of chlorination stress. Among other pathologies, acute kidney injury, glomerulonephritis, diabetic nephropathy, and bladder cancer have been associated with exposure to pathological chlorination stress (110, 162–166).

### Potential Therapeutic and Chemopreventive Opportunities of Topical HOCl With a Focus on Solar UV-Induced Skin Carcinogenesis

Remarkably, in addition to endogenous and environmental sources, skin HOCl exposure also occurs through application of topical disinfectants employed worldwide as clinical and consumer products (167–171). In human skin (as a function of concentration, pH, and exposure time), irritation and disruption of barrier function, alteration of the commensal microbiome, allergy, and contact hypersensitivity are expected outcomes of inappropriate topical HOCl product use not compliant with standard of care (**Figure 5D**) (142, 143). Also, it has been hypothesized that DBPs in drinking water correlate with risk of skin cancer (172). Importantly, HOCl-based therapeutics optimized for topical delivery are now serving as pharmaceutical formulations for wound management, scar prevention, diabetic ulcers, atopic dermatitis, pruritus, psoriasis, and seborrheic dermatitis (84, 168, 173, 174). Suppression of inflammatory gene expression with downregulation of iNOS and COX-2 downstream of HOCl-dependent IKK inactivation represents the crucial mechanistic basis underlying HOCl-dependent therapeutic efficacy targeting psoriasis and radiation dermatitis (35). The same mechanism has also been substantiated attenuating experimental melanoma progression as a result of myeloid cell-derived HOCl (175). In addition, HOCl-hydrogel formulations have shown immunotherapeutic efficacy against experimental murine melanoma (176). Consistent with these observations, a suppressive role of HOCl in the control of cancer cell viability and tumor progression has been envisioned and further substantiated (71, 177, 178).

More recently, we have investigated the molecular consequences of solar simulated ultraviolet (UV) radiation and HOCl combinations, a procedure mimicking co-exposure experienced for example by recreational swimmers exposed to both HOCl (pool disinfectant) and UV (solar radiation). First, we have profiled the HOCl-induced stress response in reconstructed human epidermis and SKH-1 hairless mouse skin (36). In AP-1 transgenic SKH-1 luciferase-reporter mice, topical HOCl suppressed UV-induced inflammatory signaling assessed by bioluminescent imaging and gene expression analysis documenting HOCl-antagonism of solar UV-induced AP-1 activation. Co-exposure studies (combining topical HOCl and UV) performed in SKH-1 hairless mouse skin revealed that the HOCl-induced cutaneous stress response blocks redox and inflammatory gene expression elicited by subsequent acute solar UV exposure. Remarkably, in the SKH-1 high-risk mouse model of UV-induced human keratinocytic skin cancer, relevant to actinic keratosis and subsequent malignant progression, topical HOCl blocked tumorigenic progression and inflammatory gene expression (*Ptgs2*, *Il19*, *Tlr4*), confirmed by immunohistochemical analysis including 3-chloro-tyrosine-epitopes.

These data illuminate the molecular consequences of HOCl-exposure in cutaneous organotypic and murine models assessing inflammatory gene expression and modulation of UV-induced carcinogenesis. However, the specific mechanistic involvement of NFκB and AP-1 in the HOCl-induced attenuation of UV-induced skin inflammatory gene expression and carcinogenesis remains to be elucidated. With relevance to cancer-directed preventive and potentially therapeutic activity, an HOCl-induced increased immunogenicity of proteins and enhanced uptake by dendritic cells have been observed (179). Likewise, activity as a natural adjuvant (through induction of adaptive immunity by HOCl-dependent oxidation of N-linked carbohydrates in glycoprotein), subsequently enhancing scavenger receptor uptake by antigen presenting cells, has been demonstrated, linking HOCl-potentiation of innate and adaptive immunity (180).

If translatable to photodamaged human skin, these observations provide novel insights on molecular consequences of chlorination stress not only relevant to environmental exposure but indicative of a potential photo-chemopreventive utility for topical intervention targeting early (actinic keratosis) and advanced stages of nonmelanoma skin cancer.

## Future Directions

Chlorination stress associated with HOCl/OCl^-^ exposure originating from innate and environmental sources has now been identified as a double-edged molecular sword, mediating essential functions in the context of innate immunity towards microbial attack and exerting effects that are either detrimental or therapeutic to human health, particularly in the context of skin anti-inflammatory and cancer photochemopreventive topical intervention. Harnessing HOCl-dependent preventive and therapeutic effects that might benefit human patients will depend on the development of novel chemical entities and advanced formulations allowing a more controlled and targeted modulation of chlorination stress (70, 181). Indeed, additional research must carefully explore dose regimens and extended release formulations that achieve anti-inflammatory and photo-chemopreventive effects while avoiding potential HOCl-induced tissue damage and irritation. In the same way, availability of specific biocompatible molecular fluorescent probes with diagnostic utility *in vitro* and *in vivo* (allowing imaging and quantitative analysis of physiological and therapeutic chlorination stress conditions) will expand our understanding of these multi-faceted versatile biochemical actors and processes as key determinants of health and disease (182).

## Author Contributions

Manuscript preparation: JS, JJ, GW. Conceptualization of work: GW. All authors contributed to the article and approved the submitted version.

## Funding

Supported in part by grants from the National Institutes of Health (1R01CA229418, 1R21ES029579, 1P01CA229112, ES007091, ES006694, and UA Cancer Center Support Grant CA023074).

## Author Disclaimer

The content is solely the responsibility of the authors and does not necessarily represent the official views of the National Cancer Institute or the National Institutes of Health.

## Conflict of Interest

The authors declare that the research was conducted in the absence of any commercial or financial relationships that could be construed as a potential conflict of interest.

## Publisher’s Note

All claims expressed in this article are solely those of the authors and do not necessarily represent those of their affiliated organizations, or those of the publisher, the editors and the reviewers. Any product that may be evaluated in this article, or claim that may be made by its manufacturer, is not guaranteed or endorsed by the publisher.

## Abbreviations

HOCl, Hypochlorous acid; UV, Ultraviolet; CPDs, Cyclobutane pyrimidine dimers; ROS, Reactive oxygen species; SABV, Sex as a biological variable; MPO, Myeloperoxidase; CBPs, Chlorination byproducts; CSAD, Cysteine sulfinic acid decarboxylase; CDO1, Cysteine dioxygenase; FIFRA, Federal Insecticide, Fungicide, and Rodenticide Act; DBP, Disinfection byproducts; PPCPs, Pharmaceuticals and personal care products; AHR, Airway hyperresponsiveness; GI, Gastrointestinal.

## References

[1] McLafferty E Hendry C Alistair F. The Integumentary System: Anatomy, Physiology and Function of Skin. Nurs Stand (2012) 27(3):35–42. doi: 10.7748/ns2012.09.27.3.35.c9299 23248884

[2] Slominski A. A Nervous Breakdown in the Skin: Stress and the Epidermal Barrier. J Clin Invest (2007) 117(11):3166–9. doi: 10.1172/JCI33508 PMC2045620 17975659

[3] Elias PM. Skin Barrier Function. Curr Allergy Asthma Rep (2008) 8(4):299–305. doi: 10.1007/s11882-008-0048-0 18606081 PMC2843412

[4] Plikus MV Van Spyk EN Pham K Geyfman M Kumar V Takahashi JS. The Circadian Clock in Skin: Implications for Adult Stem Cells, Tissue Regeneration, Cancer, Aging, and Immunity. J Biol Rhythms (2015) 30(3):163–82. doi: 10.1177/0748730414563537 PMC4441597 25589491

[5] Gallo RL. Human Skin Is the Largest Epithelial Surface for Interaction With Microbes. J Invest Dermatol (2017) 137(6):1213–4. doi: 10.1016/j.jid.2016.11.045 PMC5814118 28395897

[6] Coates M Blanchard S MacLeod AS. Innate Antimicrobial Immunity in the Skin: A Protective Barrier Against Bacteria, Viruses, and Fungi. PloS Pathog (2018) 14(12):e1007353. doi: 10.1371/journal.ppat.1007353 30522130 PMC6283644

[7] Byrd AL Belkaid Y Segre JA. The Human Skin Microbiome. Nat Rev Microbiol (2018) 16(3):143–55. doi: 10.1038/nrmicro.2017.157 29332945

[8] Schalka S Silva MS Lopes LF de Freitas LM Baptista MS. The Skin Redoxome. J Eur Acad Dermatol Venereol (2022) 36(2):181–95. doi: 10.1111/jdv.17780 34719068

[9] Wondrak GT. Let the Sun Shine in: Mechanisms and Potential for Therapeutics in Skin Photodamage. Curr Opin Investig Drugs (2007) 8(5):390–400. 17520868

[10] Brash DE Ziegler A Jonason AS Simon JA Kunala S Leffell DJ. Sunlight and Sunburn in Human Skin Cancer: P53, Apoptosis, and Tumor Promotion. J Investig Dermatol Symp Proc (1996) 1(2):136–42. 9627707

[11] Wondrak GT Jacobson MK Jacobson EL. Endogenous UVA-Photosensitizers: Mediators of Skin Photodamage and Novel Targets for Skin Photoprotection. Photochem Photobiol Sci (2006) 5(2):215–37. doi: 10.1039/B504573H 16465308

[12] Cadet J Mouret S Ravanat JL Douki T. Photoinduced Damage to Cellular DNA: Direct and Photosensitized Reactions. Photochem Photobiol (2012) 88(5):1048–65. doi: 10.1111/j.1751-1097.2012.01200.x 22780837

[13] Prasad R Katiyar SK. Crosstalk Among UV-Induced Inflammatory Mediators, DNA Damage and Epigenetic Regulators Facilitates Suppression of the Immune System. Photochem Photobiol (2017) 93(4):930–6. doi: 10.1111/php.12687 PMC5466507 27935057

[14] Appenzeller BMR Chadeau-Hyam M Aguilar L. Skin Exposome Science in Practice : Current Evidence on Hair Biomonitoring and Future Perspectives. J Eur Acad Dermatol Venereol (2020) 34 Suppl 4:26–30. doi: 10.1111/jdv.16640 32677066

[15] Grice EA Segre JA. The Skin Microbiome. Nat Rev Microbiol (2011) 9(4):244–53. doi: 10.1038/nrmicro2537 PMC3535073 21407241

[16] Juliano C Magrini GA. Cosmetic Ingredients as Emerging Pollutants of Environmental and Health Concern. A Mini-Rev Cosmetics (2017) 4(2):11. doi: 10.3390/cosmetics4020011

[17] Krutmann J Bouloc A Sore G Bernard BA Passeron T. The Skin Aging Exposome. J Dermatol Sci (2017) 85(3):152–61. doi: 10.1016/j.jdermsci.2016.09.015 27720464

[18] Ferrara F Prieux R Woodby B Valacchi G. Inflammasome Activation in Pollution-Induced Skin Conditions. Plast Reconstr Surg (2021) 147(1S-2):15S–24S. doi: 10.1097/PRS.0000000000007617 33347070

[19] Xerfan EMS Andersen ML Facina AS Tufik S Tomimori J. Sleep Loss and the Skin: Possible Effects of This Stressful State on Cutaneous Regeneration During Nocturnal Dermatological Treatment and Related Pathways. Dermatol Ther (2022) 35(2):e15226. doi: 10.1111/dth.15226 34820993

[20] Celebi Sozener Z Ozdel Ozturk B Cerci P Turk M Gorgulu Akin B Akdis M. Epithelial Barrier Hypothesis: Effect of External Exposome on Microbiome and Epithelial Barriers in Allergic Disease. Allergy (2022). doi: 10.1111/all.15240 PMC9306534 35108405

[21] Cope RB Imsilp K Morrow CK Hartman J Schaeffer DJ Hansen LG. Exposure to Soil Contaminated With an Environmental PCB/PCDD/PCDF Mixture Modulates Ultraviolet Radiation-Induced Non-Melanoma Skin Carcinogenesis in the Crl:SKH1-hrBR Hairless Mouse. Cancer Lett (2003) 191(2):145–54. doi: 10.1016/S0304-3835(02)00636-5 12618327

[22] Burns FJ Uddin AN Wu F Nadas A Rossman TG. Arsenic-Induced Enhancement of Ultraviolet Radiation Carcinogenesis in Mouse Skin: A Dose-Response Study. Environ Health Perspect (2004) 112(5):599–603. doi: 10.1289/ehp.6655 15064167 PMC1241927

[23] Wang Y Gao D Atencio DP Perez E Saladi R Moore J. Combined Subcarcinogenic Benzo[a]Pyrene and UVA Synergistically Caused High Tumor Incidence and Mutations in H-Ras Gene, But Not P53, in SKH-1 Hairless Mouse Skin. Int J Cancer (2005) 116(2):193–9. doi: 10.1002/ijc.21039 15800929

[24] Meeran SM Singh T Nagy TR Katiyar SK. High-Fat Diet Exacerbates Inflammation and Cell Survival Signals in the Skin of Ultraviolet B-Irradiated C57BL/6 Mice. Toxicol Appl Pharmacol (2009) 241(3):303–10. doi: 10.1016/j.taap.2009.09.003 19747500

[25] Magiatis P Pappas P Gaitanis G Mexia N Melliou E Galanou M. Malassezia Yeasts Produce a Collection of Exceptionally Potent Activators of the Ah (Dioxin) Receptor Detected in Diseased Human Skin. J Invest Dermatol (2013) 133(8):2023–30. doi: 10.1038/jid.2013.92 PMC3714356 23448877

[26] O'Gorman SM Murphy GM. Photosensitizing Medications and Photocarcinogenesis. Photodermatol Photoimmunol Photomed (2014) 30(1):8–14. doi: 10.1111/phpp.12085 24393207

[27] Hasche D Stephan S Braspenning-Wesch I Mikulec J Niebler M Grone HJ. The Interplay of UV and Cutaneous Papillomavirus Infection in Skin Cancer Development. PloS Pathog (2017) 13(11):e1006723. doi: 10.1371/journal.ppat.1006723 29190285 PMC5708609

[28] Godar DE. UV and Reactive Oxygen Species Activate Human Papillomaviruses Causing Skin Cancers. Curr Probl Dermatol (2021) 55:339–53. doi: 10.1159/000517643 34698023

[29] Rojo de la Vega M Zhang DD Wondrak GT. Topical Bixin Confers NRF2-Dependent Protection Against Photodamage and Hair Graying in Mouse Skin. Front Pharmacol (2018) 9:287. doi: 10.3389/fphar.2018.00287 29636694 PMC5880955

[30] Morita A Torii K Maeda A Yamaguchi Y. Molecular Basis of Tobacco Smoke-Induced Premature Skin Aging. J Investig Dermatol Symp Proc (2009) 14(1):53–5. doi: 10.1038/jidsymp.2009.13 19675554

[31] Gromkowska-Kepka KJ Puscion-Jakubik A Markiewicz-Zukowska R Socha K. The Impact of Ultraviolet Radiation on Skin Photoaging - Review of *In Vitro* Studies. J Cosmet Dermatol (2021) 20(11):3427–31. doi: 10.1111/jocd.14033 PMC8597149 33655657

[32] Martens MC Emmert S Boeckmann L. Sunlight, Vitamin D, and Xeroderma Pigmentosum. Adv Exp Med Biol (2020) 1268:319–31. doi: 10.1007/978-3-030-46227-7_16 32918226

[33] Rizza ERH DiGiovanna JJ Khan SG Tamura D Jeskey JD Kraemer KH. Xeroderma Pigmentosum: A Model for Human Premature Aging. J Invest Dermatol (2021) 141(4S):976–84. doi: 10.1016/j.jid.2020.11.012 PMC7987754 33436302

[34] Richardson SD DeMarini DM Kogevinas M Fernandez P Marco E Lourencetti C. What's in the Pool? A Comprehensive Identification of Disinfection by-Products and Assessment of Mutagenicity of Chlorinated and Brominated Swimming Pool Water. Environ Health Perspect (2010) 118(11):1523–30. doi: 10.1289/ehp.1001965 PMC2974688 20833605

[35] Leung TH Zhang LF Wang J Ning S Knox SJ Kim SK. Topical Hypochlorite Ameliorates NF-kappaB-Mediated Skin Diseases in Mice. J Clin Invest (2013) 123(12):5361–70. doi: 10.1172/JCI70895 PMC3859383 24231355

[36] Jandova J Snell J Hua A Dickinson S Fimbres J Wondrak GT. Topical Hypochlorous Acid (HOCl) Blocks Inflammatory Gene Expression and Tumorigenic Progression in UV-Exposed SKH-1 High Risk Mouse Skin. Redox Biol (2021) 45:102042. doi: 10.1016/j.redox.2021.102042 34144392 PMC8217684

[37] Kishimoto N Nishimura H. Effect of pH and Moar Ratio of Pollutant to Oxidant on a Photochemical Advanced Oxidation Process Using Hypochlorite. Environ Technol (2015) 36(19):2436–42. doi: 10.1080/09593330.2015.1034187 25809495

[38] Remucal CK Manley D. Emerging Investigators Series: The Efficacy of Chlorine Photolysis as an Advanced Oxidation Process for Drinking Water Treatment. Environ Sci Water Res Technol (2016) 2:565–79. doi: 10.1039/C6EW00029K

[39] Prutz WA. Hypochlorous Acid Interactions With Thiols, Nucleotides, DNA, and Other Biological Substrates. Arch Biochem Biophys (1996) 332(1):110–20. doi: 10.1006/abbi.1996.0322 8806715

[40] Armesto XL Canle ML Garcia MV Santaballa JA. Aqueous Chemistry of N-Halo-Compounds. Chem Soc Rev (1998) 27:453–60. doi: 10.1039/a827453z

[41] Armesto XL Canle ML Fernandez MI Garcia MV Santaballa JA. First Steps in the Oxidation of Sulfur-Containing Amino Acids by Hypohalogenation: Very Fast Generation of Intermediate Sulfenyl Halides and Halosulfonium Cations. Tetrahedron (2000) 56:1103–9. doi: 10.1016/S0040-4020(99)01066-2

[42] Pattison DI Davies MJ. Absolute Rate Constants for the Reaction of Hypochlorous Acid With Protein Side Chains and Peptide Bonds. Chem Res Toxicol (2001) 14(10):1453–64. doi: 10.1021/tx0155451 11599938

[43] Pattison DI Hawkins CL Davies MJ. Hypochlorous Acid-Mediated Oxidation of Lipid Components and Antioxidants Present in Low-Density Lipoproteins: Absolute Rate Constants, Product Analysis, and Computational Modeling. Chem Res Toxicol (2003) 16(4):439–49. doi: 10.1021/tx025670s 12703960

[44] Wahman DG. Chlorinated Cyanurates: Review of Water Chemistry and Associated Drinking Water Implications. J Am Water Works Assoc (2018) 110(9):E1–E15. doi: 10.1002/awwa.1086 PMC6178841 30319139

[45] Chuang YH Shi HJ. UV/Chlorinated Cyanurates as an Emerging Advanced Oxidation Process for Drinking Water and Potable Reuse Treatments. Water Res (2022) 211:118075. doi: 10.1016/j.watres.2022.118075 35066259

[46] Ulfig A Leichert LI. The Effects of Neutrophil-Generated Hypochlorous Acid and Other Hypohalous Acids on Host and Pathogens. Cell Mol Life Sci (2021) 78(2):385–414. doi: 10.1007/s00018-020-03591-y 32661559 PMC7873122

[47] Hawkins CL. Hypochlorous Acid-Mediated Modification of Proteins and its Consequences. Essays Biochem (2020) 64(1):75–86. doi: 10.1042/EBC20190045 31867603

[48] Davies MJ. Myeloperoxidase: Mechanisms, Reactions and Inhibition as a Therapeutic Strategy in Inflammatory Diseases. Pharmacol Ther (2021) 218:107685. doi: 10.1016/j.pharmthera.2020.107685 32961264

[49] Casciaro M Di Salvo E Pace E Ventura-Spagnolo E Navarra M Gangemi S. Chlorinative Stress in Age-Related Diseases: A Literature Review. Immun Ageing (2017) 14:21. doi: 10.1186/s12979-017-0104-5 29163665 PMC5686828

[50] Samanta S Govindaraju T. Unambiguous Detection of Elevated Levels of Hypochlorous Acid in Double Transgenic AD Mouse Brain. ACS Chem Neurosci (2019) 10(12):4847–53. doi: 10.1021/acschemneuro.9b00554 31790189

[51] Trush MA Egner PA Kensler TW. Myeloperoxidase as a Biomarker of Skin Irritation and Inflammation. Food Chem Toxicol (1994) 32(2):143–7. doi: 10.1016/0278-6915(94)90175-9 8132173

[52] Metz M Lammel V Gibbs BF Maurer M. Inflammatory Murine Skin Responses to UV-B Light are Partially Dependent on Endothelin-1 and Mast Cells. Am J Pathol (2006) 169(3):815–22. doi: 10.2353/ajpath.2006.060037 PMC1698813 16936258

[53] Meeran SM Punathil T Katiyar SK. IL-12 Deficiency Exacerbates Inflammatory Responses in UV-Irradiated Skin and Skin Tumors. J Invest Dermatol (2008) 128(11):2716–27. doi: 10.1038/jid.2008.140 PMC2574589 18509359

[54] Rijken F Bruijnzeel PL. The Pathogenesis of Photoaging: The Role of Neutrophils and Neutrophil-Derived Enzymes. J Investig Dermatol Symp Proc (2009) 14(1):67–72. doi: 10.1038/jidsymp.2009.15 19675558

[55] Gasparoto TH de Oliveira CE de Freitas LT Pinheiro CR Ramos RN da Silva AL. Inflammatory Events During Murine Squamous Cell Carcinoma Development. J Inflammation (Lond) (2012) 9(1):46. doi: 10.1186/1476-9255-9-46 PMC3542019 23176085

[56] Zawrotniak M Bartnicka D Rapala-Kozik M. UVA and UVB Radiation Induce the Formation of Neutrophil Extracellular Traps by Human Polymorphonuclear Cells. J Photochem Photobiol B (2019) 196:111511. doi: 10.1016/j.jphotobiol.2019.111511 31129510

[57] Haskamp S Bruns H Hahn M Hoffmann M Gregor A Lohr S. Myeloperoxidase Modulates Inflammation in Generalized Pustular Psoriasis and Additional Rare Pustular Skin Diseases. Am J Hum Genet (2020) 107(3):527–38. doi: 10.1016/j.ajhg.2020.07.001 PMC7477008 32758447

[58] Strzepa A Gurski CJ Dittel LJ Szczepanik M Pritchard KA Jr. Dittel BN. Neutrophil-Derived Myeloperoxidase Facilitates Both the Induction and Elicitation Phases of Contact Hypersensitivity. Front Immunol (2020) 11:608871. doi: 10.3389/fimmu.2020.608871 33569056 PMC7868335

[59] Neu SD Strzepa A Martin D Sorci-Thomas MG Pritchard KA Jr. Dittel BN. Myeloperoxidase Inhibition Ameliorates Plaque Psoriasis in Mice. Antioxid (Basel) (2021) 10(9):1338. doi: 10.3390/antiox10091338 PMC8472607 34572970

[60] Moe CL Rheingans RD. Global Challenges in Water, Sanitation and Health. J Water Health (2006) 4 Suppl 1:41–57. doi: 10.2166/wh.2006.0043 16493899

[61] Rutala WA Cole EC Thomann CA Weber DJ. Stability and Bactericidal Activity of Chlorine Solutions. Infect Control Hosp Epidemiol (1998) 19(5):323–7. doi: 10.1086/647822 9613692

[62] Daumer KM Khan AU Steinbeck MJ. Chlorination of Pyridinium Compounds. Possible Role of Hypochlorite, N-Chloramines, and Chlorine in the Oxidation of Pyridinoline Cross-Links of Articular Cartilage Collagen Type II During Acute Inflammation. J Biol Chem (2000) 275(44):34681–92. doi: 10.1074/jbc.M002003200 PMC2941819 10940296

[63] Wastensson G Eriksson K. Inorganic Chloramines: A Critical Review of the Toxicological and Epidemiological Evidence as a Basis for Occupational Exposure Limit Setting. Crit Rev Toxicol (2020) 50(3):219–71. doi: 10.1080/10408444.2020.1744514 32484073

[64] Gottardi W Debabov D Nagl M. N-Chloramines, a Promising Class of Well-Tolerated Topical Anti-Infectives. Antimicrob Agents Chemother (2013) 57(3):1107–14. doi: 10.1128/AAC.02132-12 PMC3591902 23295936

[65] Maitra D Byun J Andreana PR Abdulhamid I Saed GM Diamond MP. Mechanism of Hypochlorous Acid-Mediated Heme Destruction and Free Iron Release. Free Radic Biol Med (2011) 51(2):364–73. doi: 10.1016/j.freeradbiomed.2011.03.040 PMC3378337 21466849

[66] Maitra D Shaeib F Abdulhamid I Abdulridha RM Saed GM Diamond MP. Myeloperoxidase Acts as a Source of Free Iron During Steady-State Catalysis by a Feedback Inhibitory Pathway. Free Radic Biol Med (2013) 63:90–8. doi: 10.1016/j.freeradbiomed.2013.04.009 PMC3863623 23624305

[67] Candeias LP Stratford MR Wardman P. Formation of Hydroxyl Radicals on Reaction of Hypochlorous Acid With Ferrocyanide, a Model Iron(II) Complex. Free Radic Res (1994) 20(4):241–9. doi: 10.3109/10715769409147520 8205226

[68] Candeias LP Patel KB Stratford MR Wardman P. Free Hydroxyl Radicals are Formed on Reaction Between the Neutrophil-Derived Species Superoxide Anion and Hypochlorous Acid. FEBS Lett (1993) 333(1-2):151–3. doi: 10.1016/0014-5793(93)80394-A 8224156

[69] Miyamoto S Martinez GR Rettori D Augusto O Medeiros MH Di Mascio P. Linoleic Acid Hydroperoxide Reacts With Hypochlorous Acid, Generating Peroxyl Radical Intermediates and Singlet Molecular Oxygen. Proc Natl Acad Sci USA (2006) 103(2):293–8. doi: 10.1073/pnas.0508170103 PMC1326168 16387855

[70] Ximenes VF Ximenes TP Morgon NH de Souza AR. Taurine Chloramine and Hydrogen Peroxide as a Potential Source of Singlet Oxygen for Topical Application. Photochem Photobiol (2021) 97(5):963–70. doi: 10.1111/php.13410 33657673

[71] Bauer G. HOCl-Dependent Singlet Oxygen and Hydroxyl Radical Generation Modulate and Induce Apoptosis of Malignant Cells. Anticancer Res (2013) 33(9):3589–602. 24023284

[72] Hazen SL Hsu FF Duffin K Heinecke JW. Molecular Chlorine Generated by the Myeloperoxidase-Hydrogen Peroxide-Chloride System of Phagocytes Converts Low Density Lipoprotein Cholesterol Into a Family of Chlorinated Sterols. J Biol Chem (1996) 271(38):23080–8. doi: 10.1074/jbc.271.38.23080 8798498

[73] Hazen SL Hsu FF Mueller DM Crowley JR Heinecke JW. Human Neutrophils Employ Chlorine Gas as an Oxidant During Phagocytosis. J Clin Invest (1996) 98(6):1283–9. doi: 10.1172/JCI118914 PMC507553 8823292

[74] Hazen SL Hsu FF Gaut JP Crowley JR Heinecke JW. Modification of Proteins and Lipids by Myeloperoxidase. Methods Enzymol (1999) 300:88–105. doi: 10.1016/S0076-6879(99)00117-2 9919513

[75] Eiserich JP Cross CE Jones AD Halliwell B van der Vliet A. Formation of Nitrating and Chlorinating Species by Reaction of Nitrite With Hypochlorous Acid. A Novel Mechanism for Nitric Oxide-Mediated Protein Modification. J Biol Chem (1996) 271(32):19199–208. doi: 10.1074/jbc.271.32.19199 8702599

[76] Hawkins CL Pattison DI Davies MJ. Hypochlorite-Induced Oxidation of Amino Acids, Peptides and Proteins. Amino Acids (2003) 25(3-4):259–74. doi: 10.1007/s00726-003-0016-x 14661089

[77] Villamena FA. Chemistry of Reactive Species. In: Reactive Species Detection in Biology. Amsterdam, Netherland: Elsevier (2017). p. 13–64. doi: 10.1016/B978-0-12-420017-3.00005-0

[78] Davies MJ Hawkins CL. The Role of Myeloperoxidase in Biomolecule Modification, Chronic Inflammation, and Disease. Antioxid Redox Signal (2020) 32(13):957–81. doi: 10.1089/ars.2020.8030 31989833

[79] Hazell LJ van den Berg JJ Stocker R. Oxidation of Low-Density Lipoprotein by Hypochlorite Causes Aggregation That is Mediated by Modification of Lysine Residues Rather Than Lipid Oxidation. Biochem J (1994) 302( Pt 1):297–304. doi: 10.1042/bj3020297 8068018 PMC1137223

[80] Shabani F McNeil J Tippett L. The Oxidative Inactivation of Tissue Inhibitor of Metalloproteinase-1 (TIMP-1) by Hypochlorous Acid (HOCI) is Suppressed by Anti-Rheumatic Drugs. Free Radic Res (1998) 28(2):115–23. doi: 10.3109/10715769809065797 9645388

[81] Kang JI Jr. Neidigh JW. Hypochlorous Acid Damages Histone Proteins Forming 3-Chlorotyrosine and 3,5-Dichlorotyrosine. Chem Res Toxicol (2008) 21(5):1028–38. doi: 10.1021/tx7003486 18452314

[82] Strosova M Karlovska J Spickett CM Grune T Orszagova Z Horakova L. Oxidative Injury Induced by Hypochlorous Acid to Ca-ATPase From Sarcoplasmic Reticulum of Skeletal Muscle and Protective Effect of Trolox. Gen Physiol Biophys (2009) 28(2):195–209. doi: 10.4149/gpb_2009_02_195 19592716

[83] Cook NL Viola HM Sharov VS Hool LC Schoneich C Davies MJ. Myeloperoxidase-Derived Oxidants Inhibit Sarco/Endoplasmic Reticulum Ca2+-ATPase Activity and Perturb Ca2+ Homeostasis in Human Coronary Artery Endothelial Cells. Free Radic Biol Med (2012) 52(5):951–61. doi: 10.1016/j.freeradbiomed.2011.12.001 PMC3736816 22214747

[84] Pelgrift RY Friedman AJ. Topical Hypochlorous Acid (HOCl) as a Potential Treatment of Pruritus. Curr Derm Rep (2013) 2:181–90. doi: 10.1007/s13671-013-0052-z

[85] Gorudko IV Grigorieva DV Shamova EV Kostevich VA Sokolov AV Mikhalchik EV. Hypohalous Acid-Modified Human Serum Albumin Induces Neutrophil NADPH Oxidase Activation, Degranulation, and Shape Change. Free Radic Biol Med (2014) 68:326–34. doi: 10.1016/j.freeradbiomed.2013.12.023 24384524

[86] Ning J Lin Z Zhao X Zhao B Miao J. Inhibiting Lysine 353 Oxidation of GRP78 by a Hypochlorous Probe Targeting Endoplasmic Reticulum Promotes Autophagy in Cancer Cells. Cell Death Dis (2019) 10(11):858. doi: 10.1038/s41419-019-2095-y 31719525 PMC6851114

[87] Ulfig A Schulz AV Muller A Lupilov N Leichert LI. N-Chlorination Mediates Protective and Immunomodulatory Effects of Oxidized Human Plasma Proteins. Elife (2019) 8:e47395. doi: 10.7554/eLife.47395 31298656 PMC6650281

[88] Robins LI Keim EK Robins DB Edgar JS Meschke JS Gafken PR. Modifications of IL-6 by Hypochlorous Acids: Effects on Receptor Binding. ACS Omega (2021) 6(51):35593–9. doi: 10.1021/acsomega.1c05297 PMC8717532 34984290

[89] Ulfig A Bader V Varatnitskaya M Lupilov N Winklhofer KF Leichert LI. Hypochlorous Acid-Modified Human Serum Albumin Suppresses MHC Class II - Dependent Antigen Presentation in Pro-Inflammatory Macrophages. Redox Biol (2021) 43:101981. doi: 10.1016/j.redox.2021.101981 33940547 PMC8105673

[90] Mainnemare A Megarbane B Soueidan A Daniel A Chapple IL. Hypochlorous Acid and Taurine-N-Monochloramine in Periodontal Diseases. J Dent Res (2004) 83(11):823–31. doi: 10.1177/154405910408301101 15505230

[91] Kalogiannis M Delikatny EJ Jeitner TM. Serotonin as a Putative Scavenger of Hypohalous Acid in the Brain. Biochim Biophys Acta (2016) 1862(4):651–61. doi: 10.1016/j.bbadis.2015.12.012 PMC4820265 26699077

[92] Winterbourn CC van den Berg JJ Roitman E Kuypers FA. Chlorohydrin Formation From Unsaturated Fatty Acids Reacted With Hypochlorous Acid. Arch Biochem Biophys (1992) 296(2):547–55. doi: 10.1016/0003-9861(92)90609-Z 1321589

[93] Carr AC van den Berg JJ Winterbourn CC. Chlorination of Cholesterol in Cell Membranes by Hypochlorous Acid. Arch Biochem Biophys (1996) 332(1):63–9. doi: 10.1006/abbi.1996.0317 8806710

[94] Carr AC Vissers MC Domigan NM Winterbourn CC. Modification of Red Cell Membrane Lipids by Hypochlorous Acid and Haemolysis by Preformed Lipid Chlorohydrins. Redox Rep (1997) 3(5-6):263–71. doi: 10.1080/13510002.1997.11747122 9754324

[95] Spickett CM Jerlich A Panasenko OM Arnhold J Pitt AR Stelmaszynska T. The Reactions of Hypochlorous Acid, the Reactive Oxygen Species Produced by Myeloperoxidase, With Lipids. Acta Biochim Pol (2000) 47(4):889–99. doi: 10.18388/abp.2000_3944 11996112

[96] Thukkani AK McHowat J Hsu FF Brennan ML Hazen SL Ford DA. Identification of Alpha-Chloro Fatty Aldehydes and Unsaturated Lysophosphatidylcholine Molecular Species in Human Atherosclerotic Lesions. Circulation (2003) 108(25):3128–33. doi: 10.1161/01.CIR.0000104564.01539.6A 14638540

[97] Kawai Y Kiyokawa H Kimura Y Kato Y Tsuchiya K Terao J. Hypochlorous Acid-Derived Modification of Phospholipids: Characterization of Aminophospholipids as Regulatory Molecules for Lipid Peroxidation. Biochemistry (2006) 45(47):14201–11. doi: 10.1021/bi0610909 17115715

[98] Lessig J Fuchs B. HOCl-Mediated Glycerophosphocholine and Glycerophosphoethanolamine Generation From Plasmalogens in Phospholipid Mixtures. Lipids (2010) 45(1):37–51. doi: 10.1007/s11745-009-3365-8 19937395

[99] Schroter J Schiller J. Chlorinated Phospholipids and Fatty Acids: (Patho)physiological Relevance, Potential Toxicity, and Analysis of Lipid Chlorohydrins. Oxid Med Cell Longev (2016) 2016:8386362. doi: 10.1155/2016/8386362 28090245 PMC5206476

[100] Masuda M Suzuki T Friesen MD Ravanat JL Cadet J Pignatelli B. Chlorination of Guanosine and Other Nucleosides by Hypochlorous Acid and Myeloperoxidase of Activated Human Neutrophils. Catalysis by Nicotine and Trimethylamine. J Biol Chem (2001) 276(44):40486–96. doi: 10.1074/jbc.M102700200 11533049

[101] Hawkins CL Davies MJ. Hypochlorite-Induced Damage to DNA, RNA, and Polynucleotides: Formation of Chloramines and Nitrogen-Centered Radicals. Chem Res Toxicol (2002) 15(1):83–92. doi: 10.1021/tx015548d 11800600

[102] Macer-Wright JL Stanley NR Portman N Tan JT Bursill C Rayner BS. A Role for Chlorinated Nucleosides in the Perturbation of Macrophage Function and Promotion of Inflammation. Chem Res Toxicol (2019) 32(6):1223–34. doi: 10.1021/acs.chemrestox.9b00044 31066272

[103] Winterbourn CC. Comparative Reactivities of Various Biological Compounds With Myeloperoxidase-Hydrogen Peroxide-Chloride, and Similarity of the Oxidant to Hypochlorite. Biochim Biophys Acta (1985) 840(2):204–10. doi: 10.1016/0304-4165(85)90120-5 2986713

[104] Gottardi W Nagl M. N-Chlorotaurine, a Natural Antiseptic With Outstanding Tolerability. J Antimicrob Chemother (2010) 65(3):399–409. doi: 10.1093/jac/dkp466 20053689

[105] Siwak J Lewinska A Wnuk M Bartosz G. Protection of Flavonoids Against Hypochlorite-Induced Protein Modifications. Food Chem (2013) 141(2):1227–41. doi: 10.1016/j.foodchem.2013.04.018 23790908

[106] Shaeib F Khan SN Ali I Najafi T Maitra D Abdulhamid I. Melatonin Prevents Myeloperoxidase Heme Destruction and the Generation of Free Iron Mediated by Self-Generated Hypochlorous Acid. PloS One (2015) 10(3):e0120737. doi: 10.1371/journal.pone.0120737 25835505 PMC4383586

[107] Asahi T Wu X Shimoda H Hisaka S Harada E Kanno T. A Mushroom-Derived Amino Acid, Ergothioneine, is a Potential Inhibitor of Inflammation-Related DNA Halogenation. Biosci Biotechnol Biochem (2016) 80(2):313–7. doi: 10.1080/09168451.2015.1083396 26338495

[108] Carvalho LAC Lopes J Kaihami GH Silva RP Bruni-Cardoso A Baldini RL. Uric Acid Disrupts Hypochlorous Acid Production and the Bactericidal Activity of HL-60 Cells. Redox Biol (2018) 16:179–88. doi: 10.1016/j.redox.2018.02.020 PMC5952876 29510342

[109] Nagl M Arnitz R Lackner M. N-Chlorotaurine, a Promising Future Candidate for Topical Therapy of Fungal Infections. Mycopathologia (2018) 183(1):161–70. doi: 10.1007/s11046-017-0175-z PMC5773618 28702855

[110] Madu H Avance J Chetyrkin S Darris C Rose KL Sanchez OA. Pyridoxamine Protects Proteins From Damage by Hypohalous Acids *In Vitro* and In Vivo. Free Radic Biol Med (2015) 89:83–90. doi: 10.1016/j.freeradbiomed.2015.07.001 26159508 PMC4684779

[111] Zoete V Bailly F Vezin H Teissier E Duriez P Fruchart JC. 4-Mercaptoimidazoles Derived From the Naturally Occurring Antioxidant Ovothiols 1. Antioxidant Properties. Free Radic Res (2000) 32(6):515–24. doi: 10.1080/10715760000300521 10798717

[112] Carroll L Karton A Radom L Davies MJ Pattison DI. Carnosine and Carcinine Derivatives Rapidly React With Hypochlorous Acid to Form Chloramines and Dichloramines. Chem Res Toxicol (2019) 32(3):513–25. doi: 10.1021/acs.chemrestox.8b00363 30693765

[113] Choe JK Hua LC Komaki Y Simpson AM McCurry DL Mitch WA. Evaluation of Histidine Reactivity and Byproduct Formation During Peptide Chlorination. Environ Sci Technol (2021) 55(3):1790–9. doi: 10.1021/acs.est.0c07408 33492937

[114] Marcinkiewicz J Kontny E. Taurine and Inflammatory Diseases. Amino Acids (2014) 46(1):7–20. doi: 10.1007/s00726-012-1361-4 22810731 PMC3894431

[115] Gottardi W Nagl M. Chemical Properties of N-Chlorotaurine Sodium, a Key Compound in the Human Defence System. Arch Pharm (Weinheim) (2002) 335(9):411–21. doi: 10.1002/1521-4184(200212)335:9<411::AID-ARDP411>3.0.CO;2-D 12447914

[116] Midwinter RG Cheah FC Moskovitz J Vissers MC Winterbourn CC. IkappaB is a Sensitive Target for Oxidation by Cell-Permeable Chloramines: Inhibition of NF-kappaB Activity by Glycine Chloramine Through Methionine Oxidation. Biochem J (2006) 396(1):71–8. doi: 10.1042/BJ20052026 PMC1450002 16405428

[117] Kanayama A Inoue J Sugita-Konishi Y Shimizu M Miyamoto Y. Oxidation of Ikappa Balpha at Methionine 45 is One Cause of Taurine Chloramine-Induced Inhibition of NF-Kappa B Activation. J Biol Chem (2002) 277(27):24049–56. doi: 10.1074/jbc.M110832200 11983684

[118] Seidel U Huebbe P Rimbach G. Taurine: A Regulator of Cellular Redox Homeostasis and Skeletal Muscle Function. Mol Nutr Food Res (2019) 63(16):e1800569. doi: 10.1002/mnfr.201800569 30211983

[119] van Veldhoven K Keski-Rahkonen P Barupal DK Villanueva CM Font-Ribera L Scalbert A. Effects of Exposure to Water Disinfection by-Products in a Swimming Pool: A Metabolome-Wide Association Study. Environ Int (2018) 111:60–70. doi: 10.1016/j.envint.2017.11.017 29179034 PMC5786667

[120] Leusch FDL Neale PA Busetti F Card M Humpage A Orbell JD. Transformation of Endocrine Disrupting Chemicals, Pharmaceutical and Personal Care Products During Drinking Water Disinfection. Sci Total Environ (2019) 657:1480–90. doi: 10.1016/j.scitotenv.2018.12.106 30677914

[121] Suppes LM Abrell L Dufour AP Reynolds KA. Assessment of Swimmer Behaviors on Pool Water Ingestion. J Water Health (2014) 12(2):269–79. doi: 10.2166/wh.2013.123 24937221

[122] Srivastav AL Patel N Chaudhary VK. Disinfection by-Products in Drinking Water: Occurrence, Toxicity and Abatement. Environ Pollut (2020) 267:115474. doi: 10.1016/j.envpol.2020.115474 32889516

[123] Li XF Mitch WA. Drinking Water Disinfection Byproducts (DBPs) and Human Health Effects: Multidisciplinary Challenges and Opportunities. Environ Sci Technol (2018) 52(4):1681–9. doi: 10.1021/acs.est.7b05440 29283253

[124] Muellner MG Wagner ED McCalla K Richardson SD Woo YT Plewa MJ. Haloacetonitriles vs. Regulated Haloacetic Acids: Are Nitrogen-Containing DBPs More Toxic? Environ Sci Technol (2007) 41(2):645–51. doi: 10.1021/es0617441 17310735

[125] Bagheban M Baghdadi M Mohammadi A Roozbehnia P. Investigation of the Effective Factors on the Mutagen X Formation in Drinking Water by Response Surface Methodology. J Environ Manage (2019) 251:109515. doi: 10.1016/j.jenvman.2019.109515 31569020

[126] Soufan M Deborde M Legube B. Aqueous Chlorination of Diclofenac: Kinetic Study and Transformation Products Identification. Water Res (2012) 46(10):3377–86. doi: 10.1016/j.watres.2012.03.056 22525458

[127] Zhang R He Y Yao L Chen J Zhu S Rao X. Metformin Chlorination Byproducts in Drinking Water Exhibit Marked Toxicities of a Potential Health Concern. Environ Int (2021) 146:106244. doi: 10.1016/j.envint.2020.106244 33157379

[128] He Y Jin H Gao H Zhang G Ju F. Prevalence, Production, and Ecotoxicity of Chlorination-Derived Metformin Byproducts in Chinese Urban Water Systems. Sci Total Environ (2022) 816:151665. doi: 10.1016/j.scitotenv.2021.151665 34785232

[129] Negreira N Regueiro J Lopez de Alda M Barcelo D. Transformation of Tamoxifen and its Major Metabolites During Water Chlorination: Identification and in Silico Toxicity Assessment of Their Disinfection Byproducts. Water Res (2015) 85:199–207. doi: 10.1016/j.watres.2015.08.036 26320721

[130] Zhuang R Zabar R Grbovic G Dolenc D Yao J Tisler T. Stability and Toxicity of Selected Chlorinated Benzophenone-Type UV Filters in Waters. Acta Chim Slov (2013) 60(4):826–32. 24362986

[131] Crista DM Miranda MS Esteves da Silva JC. Degradation in Chlorinated Water of the UV Filter 4-Tert-Butyl-4'-Methoxydibenzoylmethane Present in Commercial Sunscreens. Environ Technol (2015) 36(9-12):1319–26. doi: 10.1080/09593330.2014.988184 25399819

[132] Sharifan H Klein D Morse AN. UV Filters Interaction in the Chlorinated Swimming Pool, a New Challenge for Urbanization, a Need for Community Scale Investigations. Environ Res (2016) 148:273–6. doi: 10.1016/j.envres.2016.04.002 27088731

[133] Zhang S Wang X Yang H Xie YF. Chlorination of Oxybenzone: Kinetics, Transformation, Disinfection Byproducts Formation, and Genotoxicity Changes. Chemosphere (2016) 154:521–7. doi: 10.1016/j.chemosphere.2016.03.116 27085067

[134] Wang C Bavcon Kralj M Kosmrlj B Yao J Kosenina S Polyakova OV. Stability and Removal of Selected Avobenzone's Chlorination Products. Chemosphere (2017) 182:238–44. doi: 10.1016/j.chemosphere.2017.04.125 28500968

[135] Yang P Kong D Ji Y Lu J Yin X Zhou Q. Chlorination and Chloramination of Benzophenone-3 and Benzophenone-4 UV Filters. Ecotoxicol Environ Saf (2018) 163:528–35. doi: 10.1016/j.ecoenv.2018.07.111 30077149

[136] Vile GF Rothwell LA Kettle AJ. Hypochlorous Acid Activates the Tumor Suppressor Protein P53 in Cultured Human Skin Fibroblasts. Arch Biochem Biophys (1998) 359(1):51–6. doi: 10.1006/abbi.1998.0881 9799559

[137] Zhu L Pi J Wachi S Andersen ME Wu R Chen Y. Identification of Nrf2-Dependent Airway Epithelial Adaptive Response to Proinflammatory Oxidant-Hypochlorous Acid Challenge by Transcription Profiling. Am J Physiol Lung Cell Mol Physiol (2008) 294(3):L469–77. doi: 10.1152/ajplung.00310.2007 18156441

[138] West JD Wang Y Morano KA. Small Molecule Activators of the Heat Shock Response: Chemical Properties, Molecular Targets, and Therapeutic Promise. Chem Res Toxicol (2012) 25(10):2036–53. doi: 10.1021/tx300264x PMC3472121 22799889

[139] Midwinter RG Vissers MC Winterbourn CC. Hypochlorous Acid Stimulation of the Mitogen-Activated Protein Kinase Pathway Enhances Cell Survival. Arch Biochem Biophys (2001) 394(1):13–20. doi: 10.1006/abbi.2001.2530 11566022

[140] Lane AE Tan JT Hawkins CL Heather AK Davies MJ. The Myeloperoxidase-Derived Oxidant HOSCN Inhibits Protein Tyrosine Phosphatases and Modulates Cell Signalling *via* the Mitogen-Activated Protein Kinase (MAPK) Pathway in Macrophages. Biochem J (2010) 430(1):161–9. doi: 10.1042/BJ20100082 PMC2911680 20528774

[141] Hoyle GW Svendsen ER. Persistent Effects of Chlorine Inhalation on Respiratory Health. Ann N Y Acad Sci (2016) 1378(1):33–40. doi: 10.1111/nyas.13139 27385061 PMC5063681

[142] Chia Shi Zhe G Green A Fong YT Lee HY Ho SF. Rare Case of Type I Hypersensitivity Reaction to Sodium Hypochlorite Solution in a Healthcare Setting. BMJ Case Rep (2016) 2016:bcr2016217228. doi: 10.1136/bcr-2016-217228 PMC5093827 27769978

[143] Goma A de Lluis R Roca-Ferrer J Lafuente J Picado C. Respiratory, Ocular and Skin Health in Recreational and Competitive Swimmers: Beneficial Effect of a New Method to Reduce Chlorine Oxidant Derivatives. Environ Res (2017) 152:315–21. doi: 10.1016/j.envres.2016.10.030 27835856

[144] Van Den Broucke S Pollaris L Vande Velde G Verbeken E Nemery B Vanoirbeek J. Irritant-Induced Asthma to Hypochlorite in Mice Due to Impairment of the Airway Barrier. Arch Toxicol (2018) 92(4):1551–61. doi: 10.1007/s00204-018-2161-8 29368146

[145] Thickett KM McCoach JS Gerber JM Sadhra S Burge PS. Occupational Asthma Caused by Chloramines in Indoor Swimming-Pool Air. Eur Respir J (2002) 19(5):827–32. doi: 10.1183/09031936.02.00232802 12030720

[146] Venglarik CJ Giron-Calle J Wigley AF Malle E Watanabe N Forman HJ. Hypochlorous Acid Alters Bronchial Epithelial Cell Membrane Properties and Prevention by Extracellular Glutathione. J Appl Physiol (1985) (2003) 95(6):2444–52. doi: 10.1152/japplphysiol.00002.2003 14514700

[147] Bougault V Turmel J St-Laurent J Bertrand M Boulet LP. Asthma, Airway Inflammation and Epithelial Damage in Swimmers and Cold-Air Athletes. Eur Respir J (2009) 33(4):740–6. doi: 10.1183/09031936.00117708 19129276

[148] Li JH Wang ZH Zhu XJ Deng ZH Cai CX Qiu LQ. Health Effects From Swimming Training in Chlorinated Pools and the Corresponding Metabolic Stress Pathways. PloS One (2015) 10(3):e0119241. doi: 10.1371/journal.pone.0119241 25742134 PMC4351252

[149] Font-Ribera L Kogevinas M Zock JP Gomez FP Barreiro E Nieuwenhuijsen MJ. Short-Term Changes in Respiratory Biomarkers After Swimming in a Chlorinated Pool. Environ Health Perspect (2010) 118(11):1538–44. doi: 10.1289/ehp.1001961 PMC2974690 20833607

[150] Font-Ribera L Marco E Grimalt JO Pastor S Marcos R Abramsson-Zetterberg L. Exposure to Disinfection by-Products in Swimming Pools and Biomarkers of Genotoxicity and Respiratory Damage - The PISCINA2 Study. Environ Int (2019) 131:104988. doi: 10.1016/j.envint.2019.104988 31323486

[151] Kogevinas M Villanueva CM Font-Ribera L Liviac D Bustamante M Espinoza F. Genotoxic Effects in Swimmers Exposed to Disinfection by-Products in Indoor Swimming Pools. Environ Health Perspect (2010) 118(11):1531–7. doi: 10.1289/ehp.1001959 PMC2974689 20833606

[152] Vizcaya D Christensen KY Lavoue J Siemiatycki J. Risk of Lung Cancer Associated With Six Types of Chlorinated Solvents: Results From Two Case-Control Studies in Montreal, Canada. Occup Environ Med (2013) 70(2):81–5. doi: 10.1136/oemed-2012-101155 23104733

[153] Dias MF Reis MP Acurcio LB Carmo AO Diamantino CF Motta AM. Changes in Mouse Gut Bacterial Community in Response to Different Types of Drinking Water. Water Res (2018) 132:79–89. doi: 10.1016/j.watres.2017.12.052 29306702

[154] Fish KE Reeves-McLaren N Husband S Boxall J. Unchartered Waters: The Unintended Impacts of Residual Chlorine on Water Quality and Biofilms. NPJ Biofilms Microbiomes (2020) 6(1):34. doi: 10.1038/s41522-020-00144-w 32978404 PMC7519676

[155] Sasada T Hinoi T Saito Y Adachi T Takakura Y Kawaguchi Y. Chlorinated Water Modulates the Development of Colorectal Tumors With Chromosomal Instability and Gut Microbiota in Apc-Deficient Mice. PloS One (2015) 10(7):e0132435. doi: 10.1371/journal.pone.0132435 26186212 PMC4505894

[156] El-Tawil AM. Colorectal Cancers and Chlorinated Water. World J Gastrointest Oncol (2016) 8(4):402–9. doi: 10.4251/wjgo.v8.i4.402 PMC4824718 27096035

[157] Prochazka E Melvin SD Escher BI Plewa MJ Leusch FDL. Global Transcriptional Analysis of Nontransformed Human Intestinal Epithelial Cells (FHs 74 Int) After Exposure to Selected Drinking Water Disinfection By-Products. Environ Health Perspect (2019) 127(11):117006. doi: 10.1289/EHP4945 31755747 PMC6927499

[158] Chang JH Vogt CR Sun GY Sun AY. Effects of Acute Administration of Chlorinated Water on Liver Lipids. Lipids (1981) 16(5):336–40. doi: 10.1007/BF02534958 7253842

[159] Plaa GL. Chlorinated Methanes and Liver Injury: Highlights of the Past 50 Years. Annu Rev Pharmacol Toxicol (2000) 40:42–65. doi: 10.1146/annurev.pharmtox.40.1.43 10836127

[160] Faustino-Rocha AI Rodrigues D da Costa RG Diniz C Aragao S Talhada D. Trihalomethanes in Liver Pathology: Mitochondrial Dysfunction and Oxidative Stress in the Mouse. Environ Toxicol (2016) 31(8):1009–16. doi: 10.1002/tox.22110 25640707

[161] Zheng S Yang Y Wen C Liu W Cao L Feng X. Effects of Environmental Contaminants in Water Resources on Nonalcoholic Fatty Liver Disease. Environ Int (2021) 154:106555. doi: 10.1016/j.envint.2021.106555 33857709

[162] Peck B Workeneh B Kadikoy H Patel SJ Abdellatif A. Spectrum of Sodium Hypochlorite Toxicity in Man-Also a Concern for Nephrologists. NDT Plus (2011) 4(4):231–5. doi: 10.1093/ndtplus/sfr053 PMC4421444 25949487

[163] Brown KL Darris C Rose KL Sanchez OA Madu H Avance J. Hypohalous Acids Contribute to Renal Extracellular Matrix Damage in Experimental Diabetes. Diabetes (2015) 64(6):2242–53. doi: 10.2337/db14-1001 PMC4439565 25605804

[164] Afshinnia F Zeng L Byun J Gadegbeku CA Magnone MC Whatling C. Myeloperoxidase Levels and Its Product 3-Chlorotyrosine Predict Chronic Kidney Disease Severity and Associated Coronary Artery Disease. Am J Nephrol (2017) 46(1):73–81. doi: 10.1159/000477766 28668952 PMC5560990

[165] Parvez S Ashby JL Kimura SY Richardson SD. Exposure Characterization of Haloacetic Acids in Humans for Exposure and Risk Assessment Applications: An Exploratory Study. Int J Environ Res Public Health (2019) 16(3):471. doi: 10.3390/ijerph16030471 PMC6388255 30736287

[166] Evlampidou I Font-Ribera L Rojas-Rueda D Gracia-Lavedan E Costet N Pearce N. Trihalomethanes in Drinking Water and Bladder Cancer Burden in the European Union. Environ Health Perspect (2020) 128(1):17001. doi: 10.1289/EHP4495 31939704 PMC7015561

[167] Stroman DW Mintun K Epstein AB Brimer CM Patel CR Branch JD. Reduction in Bacterial Load Using Hypochlorous Acid Hygiene Solution on Ocular Skin. Clin Ophthalmol (2017) 11:707–14. doi: 10.2147/OPTH.S132851 PMC5402722 28458509

[168] Del Rosso JQ Bhatia N. Status Report on Topical Hypochlorous Acid: Clinical Relevance of Specific Formulations, Potential Modes of Action, and Study Outcomes. J Clin Aesthet Dermatol (2018) 11(11):36–9. PMC6303114 30588272

[169] SanMiguel AJ Meisel JS Horwinski J Zheng Q Bradley CW Grice EA. Antiseptic Agents Elicit Short-Term, Personalized, and Body Site-Specific Shifts in Resident Skin Bacterial Communities. J Invest Dermatol (2018) 138(10):2234–43. doi: 10.1016/j.jid.2018.04.022 PMC6326167 29753031

[170] Severing AL Rembe JD Koester V Stuermer EK. Safety and Efficacy Profiles of Different Commercial Sodium Hypochlorite/Hypochlorous Acid Solutions (NaClO/HClO): Antimicrobial Efficacy, Cytotoxic Impact and Physicochemical Parameters In Vitro. J Antimicrob Chemother (2019) 74(2):365–72. doi: 10.1093/jac/dky432 30388236

[171] Tran AQ Topilow N Rong A Persad PJ Lee MC Lee JH. Comparison of Skin Antiseptic Agents and the Role of 0.01% Hypochlorous Acid. Aesthet Surg J (2021) 41(10):1170–5. doi: 10.1093/asj/sjaa322 PMC8438591 33247899

[172] Karagas MR Villanueva CM Nieuwenhuijsen M Weisel CP Cantor KP Kogevinas M. Disinfection Byproducts in Drinking Water and Skin Cancer? A Hypothesis. Cancer Causes Control (2008) 19(5):547–8. doi: 10.1007/s10552-008-9116-y PMC2640837 18219581

[173] Fukuyama T Ehling S Wilzopolski J Baumer W. Comparison of Topical Tofacitinib and 0.1% Hypochlorous Acid in a Murine Atopic Dermatitis Model. BMC Pharmacol Toxicol (2018) 19(1):37. doi: 10.1186/s40360-018-0232-3 29970189 PMC6029395

[174] Fukuyama T Martel BC Linder KE Ehling S Ganchingco JR Baumer W. Hypochlorous Acid is Antipruritic and Anti-Inflammatory in a Mouse Model of Atopic Dermatitis. Clin Exp Allergy (2018) 48(1):78–88. doi: 10.1111/cea.13045 29028288

[175] Liu TW Gammon ST Yang P Fuentes D Piwnica-Worms D. Myeloid Cell-Derived HOCl is a Paracrine Effector That Trans-Inhibits IKK/NF-kappaB in Melanoma Cells and Limits Early Tumor Progression. Sci Signal (2021) 14(677):eaax5971. doi: 10.1126/scisignal.aax5971 33824181 PMC12506183

[176] Zhou Y Ye T Ye C Wan C Yuan S Liu Y. Secretions From Hypochlorous Acid-Treated Tumor Cells Delivered in a Melittin Hydrogel Potentiate Cancer Immunotherapy. Bioact Mater (2022) 9:541–53. doi: 10.1016/j.bioactmat.2021.07.019 PMC8591392 34820587

[177] Bauer G. HOCl and the Control of Oncogenesis. J Inorg Biochem (2018) 179:10–23. doi: 10.1016/j.jinorgbio.2017.11.005 29156213

[178] Freund E Miebach L Stope MB Bekeschus S. Hypochlorous Acid Selectively Promotes Toxicity and the Expression of Danger Signals in Human Abdominal Cancer Cells. Oncol Rep (2021) 45(5):71. doi: 10.3892/or.2021.8022 33760187 PMC8020206

[179] Biedron R Konopinski MK Marcinkiewicz J Jozefowski S. Oxidation by Neutrophils-Derived HOCl Increases Immunogenicity of Proteins by Converting Them Into Ligands of Several Endocytic Receptors Involved in Antigen Uptake by Dendritic Cells and Macrophages. PloS One (2015) 10(4):e0123293. doi: 10.1371/journal.pone.0123293 25849867 PMC4388828

[180] Prokopowicz ZM Arce F Biedron R Chiang CL Ciszek M Katz DR. Hypochlorous Acid: A Natural Adjuvant That Facilitates Antigen Processing, Cross-Priming, and the Induction of Adaptive Immunity. J Immunol (2010) 184(2):824–35. doi: 10.4049/jimmunol.0902606 20018624

[181] Gold MH Andriessen A Bhatia AC Bitter P Jr. Chilukuri S Cohen JL. Topical Stabilized Hypochlorous Acid: The Future Gold Standard for Wound Care and Scar Management in Dermatologic and Plastic Surgery Procedures. J Cosmet Dermatol (2020) 19(2):270–7. doi: 10.1111/jocd.13280 31904191

[182] Han J Liu X Xiong H Wang J Wang B Song X. Investigation of the Relationship Between H2O2 and HClO in Living Cells by a Bifunctional, Dual-Ratiometric Responsive Fluorescent Probe. Anal Chem (2020) 92(7):5134–42. doi: 10.1021/acs.analchem.9b05604 32122121

